# CD271 activation prevents low to high-risk progression of cutaneous squamous cell carcinoma and improves therapy outcomes

**DOI:** 10.1186/s13046-023-02737-7

**Published:** 2023-07-13

**Authors:** Marika Quadri, Natascia Tiso, Francesco Musmeci, Maria I. Morasso, Stephen R. Brooks, Luca Reggiani Bonetti, Rossana Panini, Roberta Lotti, Alessandra Marconi, Carlo Pincelli, Elisabetta Palazzo

**Affiliations:** 1 DermoLAB, Department of Surgical, Medical, Dental and Morphological Science, University of Modena and Reggio Emilia, Via Del Pozzo 71, 41124 Modena, Italy; 2 Laboratory of Developmental Genetics, Department of Biology, University of Padova, Padova, Italy; 3 CellDynamics, Bologna, Italy; 4 Laboratory of Skin Biology, National Institute of Arthritis and Musculoskeletal and Skin Diseases, NIH, Bethesda, MD USA; 5 Biodata Mining and Discovery Section, National Institute of Arthritis and Musculoskeletal and Skin Diseases, NIH, Bethesda, MD USA; 6 Department of Diagnostic, Clinic and Public Health Medicine, University of Modena and Reggio Emilia, Modena, Italy

**Keywords:** Cutaneous squamous cell carcinoma, CD271, Trk receptors, 3D models, Zebrafish

## Abstract

**Background:**

Cutaneous squamous cell carcinoma (cSCC) is the second most prevalent form of skin cancer, showing a rapid increasing incidence worldwide. Although most cSCC can be cured by surgery, a sizeable number of cases are diagnosed at advanced stages, with local invasion and distant metastatic lesions. In the skin, neurotrophins (NTs) and their receptors (CD271 and Trk) form a complex network regulating epidermal homeostasis. Recently, several works suggested a significant implication of NT receptors in cancer. However, CD271 functions in epithelial tumors are controversial and its precise role in cSCC is still to be defined.

**Methods:**

Spheroids from cSCC patients with low-risk (In situ or Well-Differentiated cSCC) or high-risk tumors (Moderately/Poorly Differentiated cSCC), were established to explore histological features, proliferation, invasion abilities, and molecular pathways modulated in response to CD271 overexpression or activation in vitro. The effect of CD271 activities on the response to therapeutics was also investigated. The impact on the metastatic process and inflammation was explored in vivo and in vitro, by using zebrafish xenograft and 2D/3D models.

**Results:**

Our data proved that CD271 is upregulated in Well-Differentiated tumors as compared to the more aggressive Moderately/Poorly Differentiated cSCC, both in vivo and in vitro. We demonstrated that CD271 activities reduce proliferation and malignancy marker expression in patient-derived cSCC spheroids at each tumor grade, by increasing neoplastic cell differentiation. CD271 overexpression significantly increases cSCC spheroid mass density, while it reduces their weight and diameter, and promotes a major fold-enrichment in differentiation and keratinization genes. Moreover, both CD271 overexpression and activation decrease cSCC cell invasiveness in vitro. A significant inhibition of the metastatic process by CD271 was observed in a newly established zebrafish cSCC model. We found that the recruitment of leucocytes by CD271-overexpressing cells directly correlates with tumor killing and this finding was further highlighted by monocyte infiltration in a THP-1-SCC13 3D model. Finally, CD271 activity synergizes with Trk receptor inhibition, by reducing spheroid viability, and significantly improves the outcome of photodynamic therapy (PTD) or chemotherapy in spheroids and zebrafish.

**Conclusion:**

Our study provides evidence that CD271 could prevent the switch between low to high-risk cSCC tumors. Because CD271 contributes to maintaining active differentiative paths and favors the response to therapies, it might be a promising target for future pharmaceutical development.

**Supplementary Information:**

The online version contains supplementary material available at 10.1186/s13046-023-02737-7.

## Background

Cutaneous Squamous Cell Carcinoma (cSCC) represents the second most frequent type of skin cancer, comprising about 20% of all skin malignancies, with a rapidly increasing incidence worldwide [1, 2]. cSCC typically includes a spectrum of progressively advanced malignancies, ranging from the precursor actinic keratosis (AK) to in situ, invasive and metastatic tumor. According to the new American Joint Committee on Cancer (AJCC), the degree of cSCC differentiation determines, along with several other factors, the grade of severity of the disease, which increases from Well-Differentiated (WD) to Moderately and Poorly Differentiated (MD-PD) tumors [3, 4]. Although most cSCC can be cured by surgical excision, a considerable number of cases are diagnosed at advanced stages with local invasion and distant metastases. Despite chemo- and radiotherapy, cSCC can be quite aggressive, leading to the death of affected individuals [5]. cSCC pathogenesis is very complex and partly unknown, involving several interconnected pathways, with a high number of mutations [6, 7]. A better understanding of the mechanisms underlying the development of cSCC would allow the discovery of potential targets for novel treatments.

Neurotrophins (NTs) and their receptors, the tyrosine kinase high-affinity receptors Trk and the low-affinity neurotrophin receptor CD271 (also called p75NTR), have been shown to form a complex network implicated in several physio-pathological functions at the skin level [8–11]. In fact, while Trk receptors are preferentially expressed by keratinocyte stem cells (KSC), thus contributing to cell proliferation and survival, CD271 may work as Trk-co-receptor. Recently, we have demonstrated that CD271 characterizes a population of an “early” transit-amplifying (TA) keratinocyte, which represents the first KSC progenitor, and its expression controls the “switch” towards the early epidermal differentiation [11, 12].

In tumors, the alterations of Trk receptor signaling pathways have been associated to the pathogenesis of different types of cancer [13], leading to the clinical development of a new class of compounds targeting the NTRK (Neurotrophic Tyrosine Kinase receptor) fusion protein [14, 15]. Head and neck tumors (HNSCC) appear to express higher levels of TrkA localized throughout the entire tumor area, as compared to TrkB and TrkC [16]. On the other hand, there are contradictory data on the localization and function of CD271 in SCC. We have recently shown that CD271 is scarcely detectable in cSCC [17, 18], while it is preferentially detected in the basal layer of esophageal (ESCC) and oral SCC (OSCC), identifying a population of stem cells [19, 20]. Moreover, CD271 proteolytic cleavage is responsible for a pro-survival activity in breast cancer cells [21], whereas the induction of CD271 stimulates apoptosis in prostate and bladder cancer cells [22, 23]. Moreover, we demonstrated the existence of a feedback loop between CD271 and the transcription factors DLX3, which seems to be necessary for the maintenance of the epidermal homeostasis. Its absence appears to be involved in the pathological alterations, leading to epithelial cancer development [17], opening the question if CD271 could be a key player in the development and progression of cSCC.

With these heterogeneous findings and lack of consistent data, we reasoned that a clear evaluation of the role of CD271 in cSCC was strongly needed. Here, we demonstrate that CD271 is definitely involved in the pathogenesis of cSCC. In fact, CD271 is more expressed in WD tumors as compared to more aggressive MD/PD tumors. CD271 activation is linked with reduced cSCC proliferation and invasion ability, by promoting differentiation, in patient or cell line-derived spheroids. CD271 expression or activation reduces cSCC metastasis in zebrafish and promotes innate immunity in both in vivo and in vitro models. Finally, we present evidence that CD271 overexpression and activation significantly improve the outcome of photodynamic therapy (PTD), chemotherapy or the effect of Trk receptors inhibition in tumor spheroids.

## Methods

### Clinical sample recruitment, ethics approval and biopsy processing

cSCC biopsies were provided by the Dermatological Clinic of the hospital of Modena and the diagnosis was confirmed by the pathologists of the Department of Diagnostic, Clinic and Public Health Medicine of the University of Modena and Reggio Emilia. The use of cSCC biopsies was approved by the Ethical Committee of Area Vasta Emilia Nord (Protocol n. 184/10 and n. 353/2017) (Modena, Italy). Biopsy digestion and patient-derived cSCC cell culture methods are described in Supplementary Information. Moreover, paraffin-embedded cSCC biopsies of different stages were collected to perform histology and immunofluorescence analysis.

### Cell and spheroid cultures and treatments

SCC13 spheroids were maintained in the SCC medium, as previously described [17]. Treatment or chemical concentrations are reported in Supplementary Information. Patient-derived cells were maintained on a 3T3 feeder-layer in DMEM/HAM’SF12 complete medium and then seeded on a collagen IV coated plate in KGM medium (Lonza, Basel, Switzerland); more details in Supplementary Information.

cSCC spheroids were transduced by viral supernatant infection and/or transfected with CD271 or scrambled siRNA (Dharmacon Inc, Lafayette, CO, USA) and treated with chemotherapy, photodynamic therapy (PDT), and Trk inhibitors, as described in Supplementary Information.

THP-1 cells were grown in RPMI 1640 with 10% FBS, 2% L-Glutamine, and 1% PSA and then maintained in DMEM with 10% FBS, 2% L-Glutamine, and 1% PSA for the experiments.

### Cutaneous SCC Skin equivalent

SCC reconstructs were obtained by seeding SCC13 cell lines on dermal equivalents, generated by human primary dermal fibroblasts induced type I collagen contraction, as previously reported [12]. Briefly, for dermal reconstructs, 0.5 ml of a cell-free collagen solution was added to tissue culture inserts (Transwell, Costar, Cambridge, MA) in 12-well plates. This precoated layer was overlaid with fibroblasts mixed with collagen type I solution (15 × 10^4^ cells/ml). After 4 days of incubation at 37 °C, 25 × 10^4^ of SCC13 mock or CD271 cells were seeded on dermal reconstructs and maintained in the submerged conditions in the SCC medium for 4 days. Finally, skin reconstructs were maintained in air-exposed conditions for 12 days before being processed for subsequent analysis.

### Real-time PCR and Western blotting

Total RNA was extracted by PureLink RNeasy Mini Kit (Invitrogen, Carlsbad, CA, USA) as indicated by the manufacturer’s instruction. 1 µg of total RNA was retrotranscribed and cDNA was used for qPCR, as previously reported [24]. Primer sequences are reported in Supplementary Table 1.

Total proteins were run on SDS–PAGE gel, transferred onto a nitrocellulose membrane, and incubated with primary antibodies, listed in Supplementary Table 2. Membranes were incubated with secondary antibodies, goat anti-mouse or goat anti-rabbit (1:3000; Bio-Rad Laboratories, Hercules, CA, USA). Bands were visualized with a chemiluminescence detection system (Amersham Biosciences UK Limited, Little Chalfont Buckinghamshire, UK).

### MTT Assay

cSCC spheroids were incubated with 0.5% MTT (3-(4,5-dimethylthiazol-2-yl)-2,5-diphenyltetrazolium bromide, Sigma-Aldrich, St. Louis, Missouri, USA) prior the end of the specific time point. Reaction is indicated in Supplementary Information.

### Histology, immunofluorescence, and immunohistochemistry

Paraffin-embedded SCC biopsies were provided from the pathological unit of the Department of Diagnostic, Clinic and Public Health Medicine of the University of Modena and Reggio Emilia. cSCC spheroids were fixed with 4% paraformaldehyde (PFA) for 30’ at RT and embedded in paraffin cSCC tissue or spheroids or skin reconstruct histology and staining (immunohistochemistry or immunofluorescence) were performed as previously described [12]. For all experiments, primary antibodies are indicated in Supplementary Table 2. Micrographs were taken on a Confocal Scanning Laser Microscopy (Leica TCS4D, Leica, Exton, PA, USA).

### FACS analysis

For CD271 evaluation by FACS analysis, SCC13 cells were treated with cisplatin (5 μg/ml) or DMSO. At 24 h, cells were detached and labeled as previously described [25]. Cells were analyzed using an Epics XL flow cytometer (Beckman Coulter). Primary antibodies are indicated in Supplementary Table 2.

### Live/dead assay and propidium iodide assay

Mock or CD271 transduced SCC13 spheroids were incubated for 30’ with a solution of Calcein AM/Ethidium Bromide (Thermo-Fisher Waltham, Massachusetts, USA). Micrographs were taken on a Confocal Scanning Laser Microscopy (Leica TCS4D, Leica, Exton, PA, USA).

SCC spheroids were washed twice in PBS and incubated for 20’ at RT with a solution of Propidium Iodide (PI, 10 mg/ml; diluted 1:100 in PBS; Sigma-Aldrich, Missouri, USA), as previously reported [24].

### Expression profiling, computational analysis, and accession number

RNA sequencing was performed on CD271 and mock SCC13 spheroids at the NIAMS Genome Core Facility at the NIH (USA), as indicated in Supplementary information. Raw data have been deposited in the Gene Expression Omnibus (GEO) site with the accession number GSE178845.

### Measurement of weight, diameter, and mass density of spheroids with W8™

Mock or CD271 SCC13 spheroids were analyzed with W8 by CellDynamics (Bologna, Italy). The physical cytometer tracks the sample during its free-falling motion throughout the vertical analysis channel of the core W8 chip, with the flow at rest [26]. Leveraging on Stokes’ law, the system measures the sample terminal velocity during its free-fall to extract mass density and weight values. More details in Supplementary Information.

### Collagen I invasion assay

SCC spheroids were implanted in a matrix of human dermal fibroblasts-collagen type I solution, as previously indicated [24]. Cell spreading after treatment was monitored according to the specific experiment, as indicated in Supplementary Information.

### Spheroids pictures analysis

Spheroid analysis Pictures of SCC spheroids were analyzed using ImageJ software (Wayne Rasband, NIH, Bethesda, MD, USA), as previously reported [25, 26].

### Zebrafish xenotransplant, whole mount in situ hybridization and immune system ablation

Zebrafish embryos were obtained from natural spawning of the nacre (*mitfa*^*w2/w2*^) fish line, *Tg(mpeg1:mCherry)g123* or *Tg(lysC:DsRed2)nz50* transgenic strains, under local Ethics Committee approval (Aut n. 407/2015-PR). SCC13 cells were stained with Vybrant Cell-Labeling Solution (Thermo-Fisher Waltham, Massachusetts, USA), as previously reported [24, 27]. Metastases were quantified as previously reported [24, 27]. Briefly, animals were monitored and observed under a fluorescent dissecting microscope by an expert in zebrafish anatomy. Animals were then classified based on the metastatic status as follows: 1) in place, animals where cells were confined to the site of injection; 2) initial metastases, cells spread from the yolk to near organs (heart, swim bladder and pharynx); 3) full metastases, animals with cell in distant organs, such as brain, skeletal muscle, and tail. Imaging was performed by Leica MZFLIII dissecting microscope equipped with a Leica DFC7000T camera.

For cisplatin and/or β-Amyloid treatments, cisplatin (5 μg/ml) was added directly to the fish water 1 day after SCC13 cell injection (1 dpi; 1 day post-injection), while β-Amyloid was injected in the zebrafish yolk at 2 dpi.

For *mpeg1.1* whole mount in situ hybridization, zebrafish were fixed in 4% PFA and the experiments were performed as previously reported [28]. To generate the *mpeg1.1* digoxigenin antisense riboprobes we used the following primers: mpeg1.1-F: 5’-CGGCGCTAACTTCTTTGACA-3’ and mpeg1.1-R: 5’-GAACGGTGGAAGACGAACTG-3’.

Macrophage ablation was obtained by injecting L-Leucyl L-Leucine methyl esther compound (L-LME; 20 mM L1002, Sigma-Aldrich), as previously reported [29]. L-LME was injected twice: immediately after cell injection (at 2 dpf), and 2 dpi/4 dpf.

### Generation of spheroid-monocyte co-culture

SCC13 cells were stained with Vybrant DIO Cell-Labeling Solution (green) (Thermo-Fisher Waltham, Massachusetts, USA) and seeded to generate spheroids. cSCC spheroids were transduced by infection with Mock or CD271 viral vectors as described above. After 72 h, THP-1 cells were stained with Vybrant Cell-Labeling Solution (red) (Thermo-Fisher Waltham, Massachusetts, USA) and co-cultured with cSCC spheroids (THP-1 was seeded at density of 10.000 cells/well). Pictures were made after 24 and 72 h from the THP-1 seeding. THP-1 infiltrating area/SCC13 spheroid area ratio was measured by ImageJ software.

### Statistical analysis

Multiparametric T-test or two-way ANOVA was performed by GraphPad Prism 9 (GraphPad Software, La Jolla, California, USA, www.graphpad.com was used). Significant p-values are indicated with * for 0.01 < *p* < 0.05, ** for 0.001 < *p* < 0.01, *** for 0.0001 < *p* < 0.001, or **** for* p* < 0.0001.

## Results

### CD271 is more expressed in less aggressive and well-differentiated cSCC

CD271 and TrkA have been recently shown to be markedly co-expressed in HNSCC, being predictive of a worse outcome in perineural invasion [30]. We have previously defined CD271 expression in different cell lines from cutaneous and oral SCC, such as SCC12B, SCC13, and SCC15, showing that its expression correlates with SCC spheroid cohesiveness [17]. However, a clear evaluation of the expression and function of the NT receptors, specifically of CD271, in cSCC is still missing.

Our analysis of the previously published dataset GSE45216 [31], aimed at identifying key differences between actinic keratosis (AK) and WD or MD/PD cSCC sample biopsies. Analysis by GEO2R revealed that CD271 mRNA expression decreases with tumor progression, with CD271 lowest mRNA level found in MD/PD tumors (Fig. S1a).

**Fig. 1:**
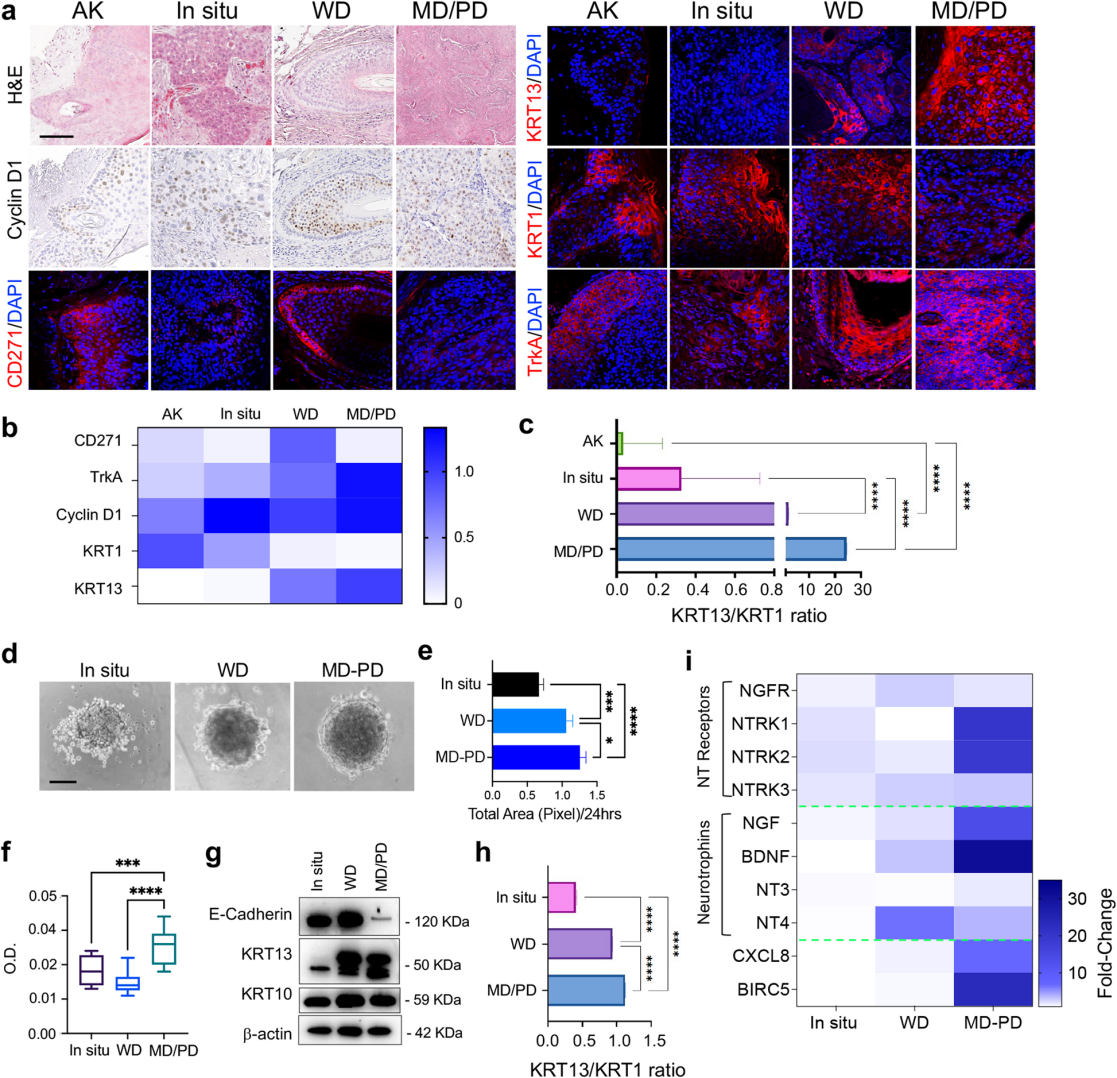
CD271 expression in cSCC biopsies and spheroids **a** Hematoxylin and Eosin (H&E) staining of AK, in situ, WD and MD/PD cSCC biopsies. CD271, Cyclin D1, TrkA, KRT1 and KRT13 expression evaluated by immunohistochemistry or immunofluorescence. Scale bar 100 µm. **b** Immunohistochemistry and immunofluorescence staining scored as follow: 0 (no cells; positivity to 20% of cells positivity), 0.1–0.75 (30–50% of cells positivity; 1–1.5 (60% to 100% of cells positivity). **c** Graphical representation of the KRT13/KRT1 intensity ratio. **d** Representative pictures of in situ, WD, and MD/PD spheroids. Scale bar = 100 μm. **e** Spheroid total area measured by ImageJ software and **f** spheroid viability evaluated by MTT assay. **g** Expression of E-cadherin, KRT13, and KRT10 evaluated in patient-derived spheroids by western blotting. β-actin was used as control. **h** Graphical representation of KRT13 and KRT10 relative protein expression ratio. **i** NTs and NTRs mRNA expression evaluated in patient-derived spheroids by qPCR. Heatmap created by Prism Graph pad software. β-actin was used as a housekeeping gene. For all experiments, the results are shown as mean ± SD of three independent experiments. Statistical analysis was performed using the two-way ANOVA. *0.01 < *p* < 0.05, ***0.0001 < *p* < 0.001, ****p < 0.0001

To validate these results and correlate CD271 expression with the tumor stage, we evaluated CD271 protein expression in human biopsies of AK or in situ, WD or MD/PD cSCC, together with differentiation (Keratin 1 – KRT1) [32] and proliferative and aggressiveness markers (Cyclin D1, Keratin 13 – KRT13, and TrkA) [30, 33, 34] (Fig. 1a). The patients’clinical features are listed in Supplementary Table 3. As expected, KRT1 expression decreases in MD/PD cSCC compared to less aggressive and more differentiated tumors, while MD/PD cSCC displayed the highest levels of Cyclin D1, TrkA, and KRT13 (Fig. 1b and Fig. S1b). Moreover, as compared to AK and in situ cSCC, WD and MD/PD tumors showed a progressively higher KRT13/KRT1 ratio, which indicates a more aggressive phenotype [35], with MD/PD being the most malignant cSCC (Fig. 1c). CD271 is more highly expressed in WD tumors, while it is not detectable in MD/PD cSCCs (Fig. 1b and Fig. S1b). Detailed examination showed that CD271 is not expressed in the more proliferative and aggressive cells that show the highest levels of Cyclin D1, KRT13 and TrkA (Fig. 1a).

In order to provide a functional analysis of these findings, we optimized and generated patient-derived cSCC human spheroids of different stages, classified, and hereafter indicated, as in situ, WD and MD/PD (Fig. 1d). Tumor features were assessed by the evaluation of spheroid’s growth and proliferative ability in vitro*,* and the expression of E-Cadherin, a negative marker of the Epithelial to Mesenchymal Transition (EMT) [36], KRT13, and the differentiation marker Keratin 10 (KRT10). As expected, MD/PD spheroids have more proliferative capacity than in situ and WD spheroids, indicating a more aggressive phenotype (Fig. 1e-f). MD/PD spheroids also express higher levels of KRT13, while the KRT13-KRT1 ratio is significantly higher in MD/PD than in the other cSCC. Noteworthy, in MD/PD cSCC E-Cadherin is barely detectable (Fig. 1g-h and Fig. S1c). These results confirm that our model maintained the characteristics of the tumor in vivo.

NTs and NT receptors (NTRs) were also evaluated in patient-derived spheroids. NTs and NTRs mRNA are variably expressed in patient-cSCC spheroids of different stages. In particular, TrkA (*NTRK1*) and TrkB (*NTRK2*) receptor mRNAs are markedly expressed in MD/PD cSCC, while CD271 mRNA (*NGFR*) is slightly detectable in WD tumors and barely detectable in MD/PD cSCC, that in turn display high levels of Survivin (*BIRC5)*, which is predictive of a poor outcome in epithelial tumors [37], and of the chemokine CXCL-8, whose expression is associated with cancer progression [38] (Fig. 1i).

These data demonstrate that the modulation of the NT network may influence cSCC development, and, more importantly, suggests that CD271 could play a preventive role against the de-differentiation stage during cSCC progression.

### CD271 overexpression decreases cSCC spheroid proliferation and viability by triggering differentiation

**Fig. 2:**
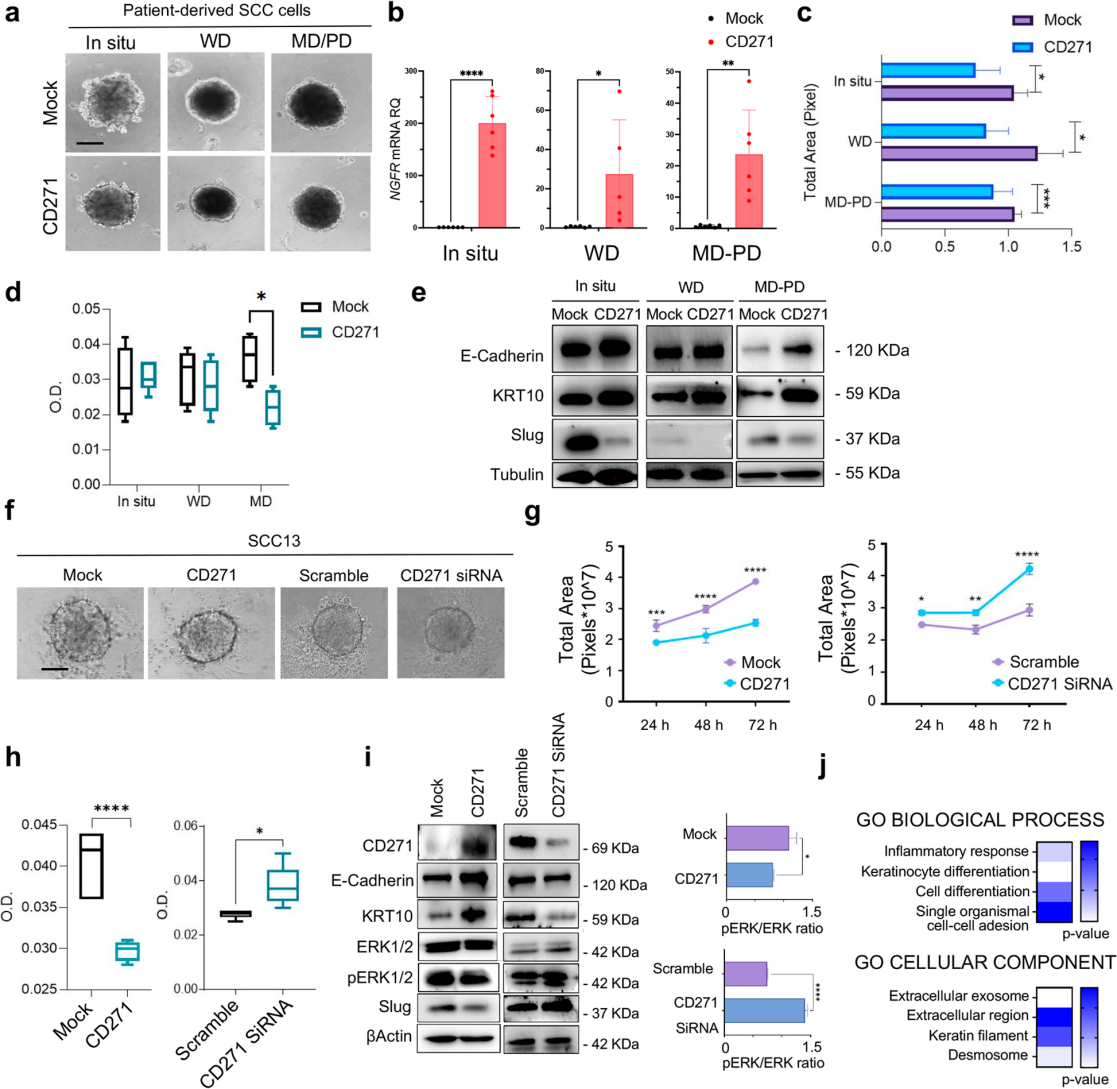
CD271 modulation affects cSCC spheroid phenotype **a** Representative picture of in situ, WD, or MD/PD patient-derived CD271-transduced or mock spheroids. Scale bar = 100 μm **b** CD271 expression by qPCR. β-actin was used as housekeeping gene. **c** Spheroid total area measured by ImageJ software. **d** Spheroid viability evaluated by MTT assay. **e** E-cadherin, KRT10, and Slug expression evaluated in patient-derived spheroids by western blotting. Tubulin was used as loading control. **f** Representative picture of SCC13 spheroids treated as described. Scale bar = 100 μm **g** Spheroid total area measured by ImageJ software. **h** Spheroid viability evaluated by MTT assay. **i** CD271, E-cadherin, KRT10, ERK1/2, pERK1/2, and Slug expression evaluated in CD271-overexpressing or silenced SCC13 spheroids by western blotting. pERK1/2/ERK1/2 expression ratio analyzed by ImageJ software. β-actin was used as loading control. **j** GO Biological Process term fold-Enrichment of RNA-seq data of CD271-transduced SCC13 spheroids vs mock determined by PANTHER Tools (a triplicate for *n* = 80 mock or CD271 spheroids). For all experiments, the results are shown as mean ± SD of three independent experiments. Statistical analysis was performed using the two-way ANOVA or multiparametric T-test. *0.01 < *p* < 0.05, **0.001 < *p* < 0.01, ***0.0001 < *p* < 0.001, ****p < 0.0001

Because CD271 modulates cell proliferation and survival in different cellular contexts [39], and promotes human keratinocyte differentiation [12], we investigated the effect of CD271 overexpression on patient-derived cSCC spheroids of different stages of aggressiveness (Fig. 2a-b). CD271 overexpression significantly reduces the area and viability of patient-derived spheroids, particularly in MD/PD spheroids (Fig. 2c-d). Interestingly, CD271-overexpressing spheroids exhibit increased protein amounts of E-Cadherin and KRT10, as compared to mock cells. On the other hand, CD271 overexpression significantly reduces the expression of the invasion marker Slug [40], suggesting that CD271 overexpression potentially reverts the aggressive phenotype of the cSCC cells (Fig. 2e and Fig. S2a).

Given the high challenge of obtaining cSCC biopsies, the subsequent functional experiments were performed on SCC cell lines of different sources, such as the cutaneous SCC13 and SCC12B cell lines and the mucous SCC15 cell lines, which have been shown to be efficient models for SCC [27]. Therefore, we assessed NTs and NT receptors (NTRs) mRNA expression in SCC cell lines derived spheroids, confirming that the neurotrophin network is operational in this tumoral setting (Fig. S2b).

**Fig. 3:**
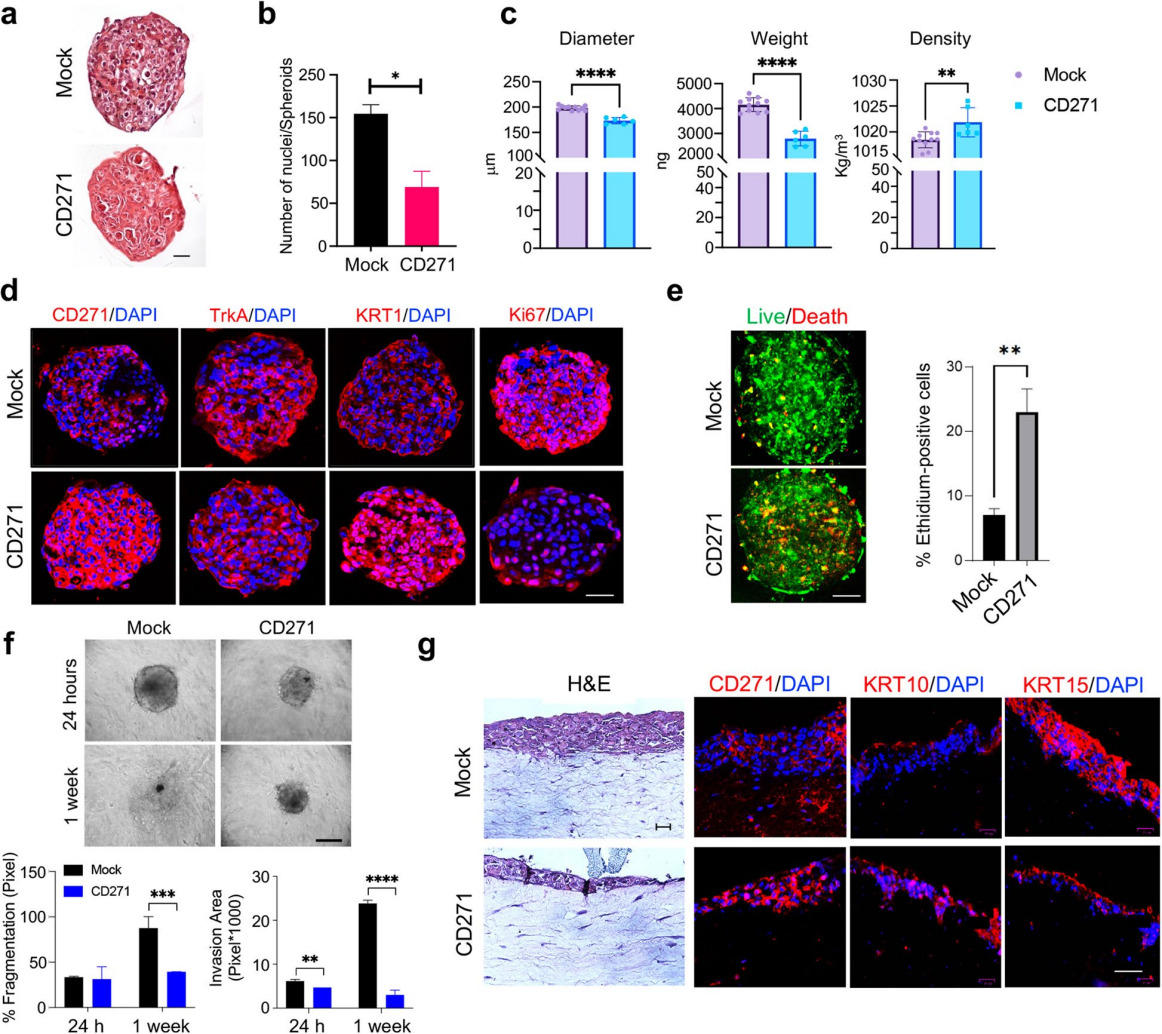
CD271 overexpression promotes cSCC differentiation **a** CD271 and mock spheroids were fixed with 4% PFA and embedded in paraffin. Spheroid histology was evaluated by Hematoxylin and Eosin (H&E) staining (scale bar = 50 μm) and **b** the number of nuclei was measured by ImageJ software. **c** Density, Weight, and Diameter of SCC13-Spheroids measured by W8™. A minimum of 10 single spheroids was analyzed for each test condition and values were extrapolated from at least 10 repetitions. **d** The expression of CD271, TrkA, KRT1 and Ki67 were evaluated in mock vs CD271-overexpressing SCC13-spheroids by immunofluorescence. Nuclei were stained with DAPI. **e** Left panel: Viability and apoptosis were measured in CD271 vs mock spheroids by LIVE/DEAD® assay (Calcein: Green, Ethidium Bromide Red). Right panel: percentage of Ethidium Bromide positive cells determined by ImageJ software analysis of micrographs. Scale bar = 50 μm **f** CD271 and mock spheroids implanted into collagen I and dermal fibroblast matrix and monitored for 2 weeks (scale bar = 100 μm). % of fragmentation and invasion area was determined by ImageJ software analysis. **g** H&E and CD271, KRT10, and KRT15 expression evaluated by immunofluorescence of mock and CD271-overexpressing SCC13-derived skin reconstruct. Nuclei were stained with DAPI. Statistical analysis was performed using the two-way ANOVA and multiparametric T-test. *0.01 < *p* < 0.05, **0.001 < *p* < 0.01, ***0.0001 < *p* < 0.001, ****p < 0.0001. Scale bar = 50 µm

Subsequently, CD271 was overexpressed or silenced in SCC13 spheroids (Fig. 2f). We observed a significantly reduced cell growth in CD271-overexpressed spheroids vs control (mock), while CD271 silencing significant increase spheroids area and viability (Fig. 2g-h). We supported the same results by using SCC12B and SCC15-derived spheroids (Fig. S2c-d). This data was subsequently confirmed by analyzing the expression of several proliferation, aggressiveness, and differentiation-associated proteins (Fig. 2i and Fig. S3a). We found an increase of the proliferative (pERK1/2, pERK/ERK ratio) and aggressiveness markers (Slug) in CD271-silenced vs scramble spheroids, while they were reverted by CD271 overexpression. Conversely, the expression of E-Cadherin and KRT10 significantly increase after CD271 overexpression, while it was significantly lower in CD271-silenced spheroids vs scramble. The altered differentiation state of CD271-overexpressing spheroids was also confirmed by RNAseq analysis, which revealed a significant Fold-enrichment of genes involved in Epidermal-cell-differentiation and Keratinization Biological-Processes (Figs. 2j and 3b).

To further support our findings, we performed a histological evaluation of CD271-overexpressing spheroids (Fig. 3a). With respect to CD271-overexpressing spheroids, control spheroid shows a greater cell density, as indicated by the high number of nuclei (Fig. 3b). Using the W8 Physical Cytometry, which allows the detection of the terminal velocity of a free-falling spheroid into a flow-channel [26], we evaluated spheroid mass density, weight, and size. CD271-overexpressing spheroids are more compact, with a smaller size and weight, as compared to controls. Moreover, they display a greater density, potentially due to the increase in extracellular components, rather than in cell number (Fig. 3c), as previously shown by histological evaluation (Fig. 3a). Moreover, the expression of TrkA and Ki67, markers of proliferation and aggressiveness that correlate with poor patients’ prognosis [16, 41], are substantially reduced in CD271-overexpressing spheroids (Fig. 3d and Fig. S4a). On the other hand, CD271-overexpressing spheroids exhibited higher levels of KRT1, indicating increased differentiation (Fig. 3d and Fig. S4a). To evaluate if CD271 overexpression also influences cell death, we performed Calcein-AM/Ethidium homodimer test on SCC13-derived spheroids, after transduction with CD271 or an empty vector. The number of apoptotic cells is increased in CD271-transduced SCC-spheroids (Fig. 3e), proving that a higher level of CD271 could potentially induce apoptosis by favoring the NT receptor balance towards a CD271-dependent signal, as previously indicated by the decrease of TrkA receptor expression in CD271-overexpressing spheroids (Fig. 3d and Fig. S4a).

To demonstrate the effect of CD271 on the aggressiveness of cSCC cells, we evaluated SCC13 cell invasion ability within a human dermal fibroblasts-collagen I matrix [42]. CD271 overexpression reduces the degree of invasion and spreading and improves circularity and compactness of spheroids. In detail, CD271-overexpressing spheroids display a lower percentage of fragmentation (% frag), which is linked to cell spreading, and a smaller invasion area that is evident at 24 h and clearly marked after 1 week (Fig. 3f).

Finally, by using SCC13 skin reconstruct, CD271-overexpression significantly reduces epidermal thickness, promotes the upregulation of KRT10, and the downregulation of Keratin-15 (KRT15), which is usually associated with proliferative keratinocytes in healthy skin [43] (Fig. 3g and Fig. S4b).

Overall, these data indicate that CD271 prevents cSCC progression, by inducing proliferation break and differentiation in cancer keratinocytes.

### CD271 overexpression abolishes cSCC metastasis in the zebrafish model

**Fig.4:**
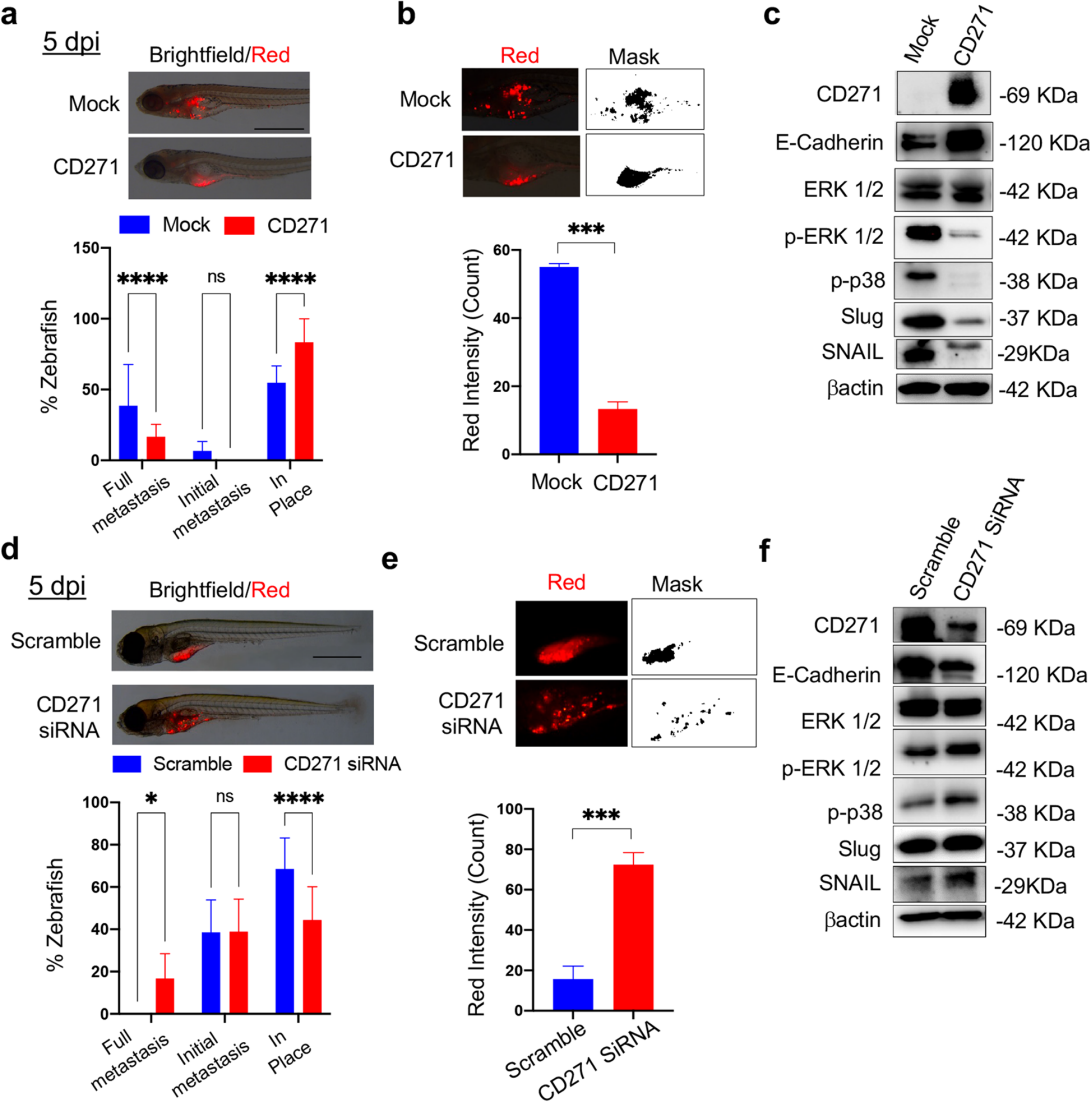
CD271 expression abolishes cSCC metastasis in zebrafish **a** Above panel: Zebrafish injected with mock or CD271-overexpressing fluorescent stained SCC13 cells (red; about 50 cells/embryo) at 5 dpi (day post-injection). Scale bar = 500 µm. Bottom panel: Metastases were quantified and classified as Full metastases, Initial metastases, and In place. **b** Above panel: Representative detail of fluorescent cSCC cells (red) and ImageJ mask. Bottom panel: Quantification of the red fluorescent intensity of cell mass in zebrafish by ImageJ software. **c** Expression of CD271, E-Cadherin, ERK1/2, pERK1/2, p-p38, Slug and Snail in cell injected into zebrafish. βactin was used as loading control. **d** Above panel: Zebrafish injected with scramble or CD271-silenced fluorescent stained SCC13 cells (red; about 50 cells/embryo) at 5 dpi (day post-injection). Scale bar = 500 µm. Bottom panel: Metastasis quantification as in (**a**). **e** Above panel: Representative detail of fluorescent cSCC cells in red and ImageJ mask. Bottom panel: quantification of the red fluorescent intensity of cell mass in zebrafish by ImageJ software. **f** Expression of CD271, E-Cadherin, ERK1/2, pERK1/2, p-p38, Slug and Snail in cell injected into zebrafish. βactin was used as loading control. Results are shown as the mean percentage of three independent experiments and a minimum of 20 injected zebrafish for each condition was used. Statistical analysis was performed using the two-way ANOVA and multiparametric T-test. *0.01 < *p* < 0.05, ***0.0001 < *p* < 0.001, ****p < 0.0001

The zebrafish (*Danio rerio*) model has recently emerged as a new tool for cancer research [27, 44]. We defined for the first time its use for cSCC, with the aim of elucidating the role of CD271. Fluorescent CD271-overexpressing or silenced SCC13 cells were injected into transparent zebrafish larvae and cell spreading was evaluated according to the scheme in Fig. S5a. No differences in fish viability are observed in zebrafish injected with CD271-overexpressing or silenced cells, as compared to control (Fig. S5b). CD271 overexpression abolishes full metastases and reduces the initial metastases, while increasing the percentage of “in place” cells (Fig. 4a and Fig. S5c). To verify if there had also been a reduction of the cells mass, fluorescence intensity, emitted by labeled cells, was measured by ImageJ software. Fluorescence intensity in zebrafish injected with CD271-overexpressing cells is significantly reduced as compared to mock cells, suggesting a CD271-induced decrease in cell proliferation (Fig. 4b). In addition, we evaluated by western blot the characteristics of the cells injected in zebrafish, confirming the same features of the 3D model (Fig. 2i). CD271-overexpressing cells injected in zebrafish express significantly decreased levels of pERK1/2 and p-p38 as well as of Slug and Snail, usually associated with cell proliferation [44, 45], and EMT [46], respectively. Conversely, CD271 overexpression significantly increases the expression of E-Cadherin (Fig. 4c and Fig. S5d).

On the other hand, the injection of CD271-silenced cells lead to the development of full metastases as compared to controls (Fig. 4d and Fig. S5c) and an increase in cell fluorescent intensity (Fig. 4e), suggesting a high proliferation rate in CD271-silenced cells, as compared to scramble cells. This finding is corroborated by the increase of pERK1/2, p-p38, Slug, and Snail expression and the decrease in E-Cadherin in CD271-silenced cells (Fig. 4f and Fig. S5d).

Altogether, these results strongly support that the upregulation of CD271 is instrumental in inhibiting the invasiveness of cSCC cells in vivo.

### CD271 overexpression improves PDT or chemotherapy outcome in cSCC

Although surgical resection is the usual standard of care for cSCC, in more advanced cases, radiation, chemotherapy and immunotherapy are required. In non-invasive forms of cSCC, when surgery is not feasible, cryotherapy, local chemotherapy (5-Fluorouracil; 5FU) and photodynamic therapy (PDT) are used [3, 6]. In particular, PDT represents one of the first therapeutic options in all spectrums of cSCC, consisting in the application of a photosensitizer that will be activated by light exposure, thus generating toxic reactive oxygen species [45].

**Fig. 5:**
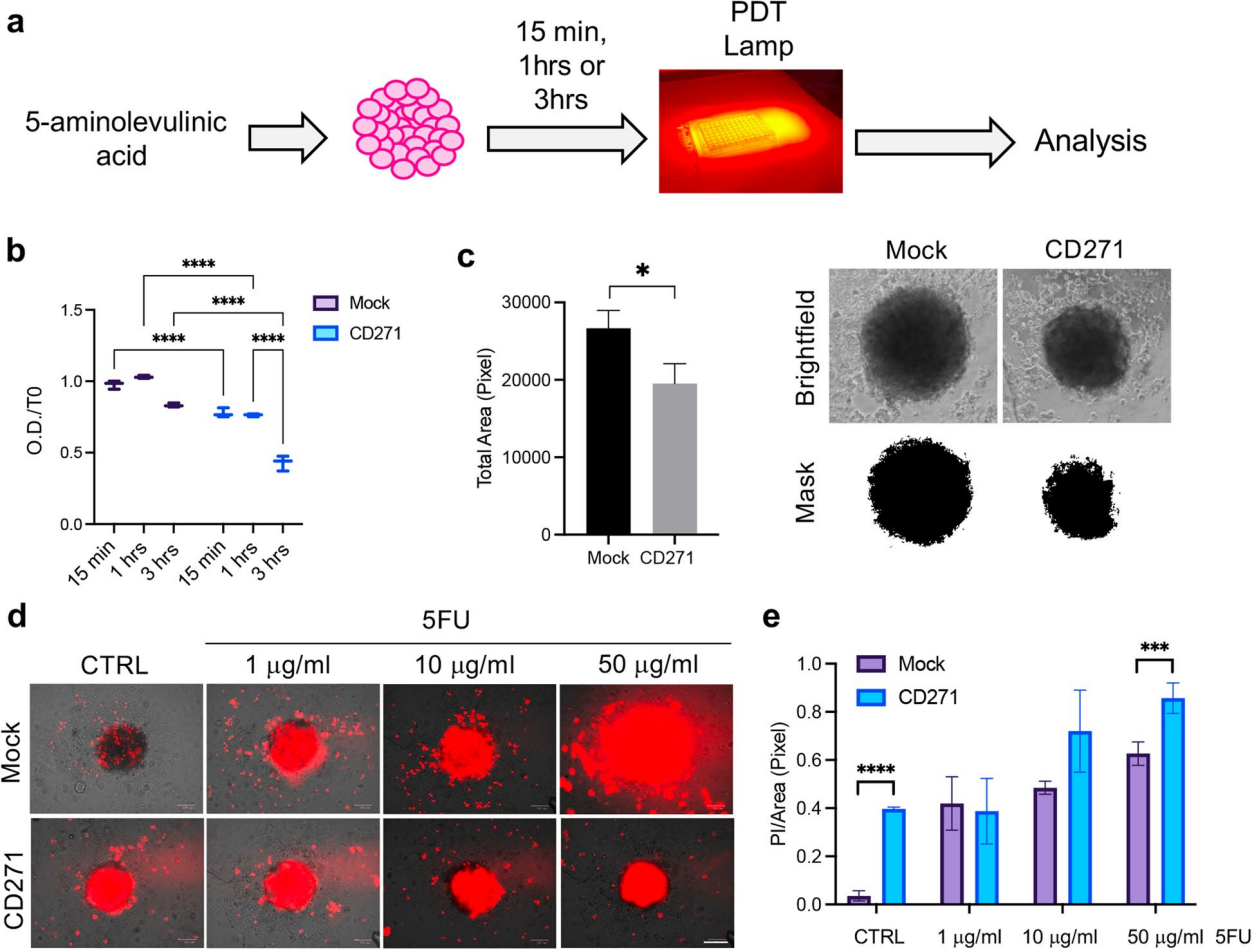
CD271 modulation improves PDT or chemotherapy efficiency **a** Schematic representation of photodynamic therapy (PDT) on SCC spheroids. **b** Cell viability evaluated at 24 h in mock vs CD271-overexpressing SCC13-derived spheroids treated with PDT for 15 min, 1 h or 3 h (*n* = 3). **c** Left panel: Spheroids total area, corresponding to 3 h PDT treatment, evaluated at 24 h. Right panel: representative brightfield images and ImageJ mask of mock and CD271-overexpressing spheroids **d** Representative images for PI staining (red)/brightfield of mock vs CD271 spheroids after treatment with 5FU (1, 10 and 50 μg/ml) or DMSO (control) at 48 h. Scale bar = 100 μm **e** Histogram representing PI staining/total spheroid area ratio (for each condition, *n* = 3). Results are shown as mean ± SD. Statistical analysis was performed using the two-way ANOVA and multiparametric t-test. *0.01 < *p* < 0.05, ***0.0001 < *p* < 0.001, ****p < 0.0001

To evaluate the effect of CD271 on cSCC therapeutic response, CD271-overexpressing spheroids were treated with PDT or 5-FU. For PDT, spheroids were treated with aminolevunic acid (Metvix®) for 15 min, 1 or 3 h before exposure to Aktilite CL128 lamp (Fig. 5a). CD271 overexpression significantly reduces cell viability in PDT-treated SCC spheroids, as compared to mock spheroids, at 24 h (Fig. 5b-c). Additionally, CD271-overexpressing spheroids were treated with 5-FU for 48 h. CD271 overexpression combined with exposure to 5-FU induces higher dose-dependent cell death as compared to mock spheroids (Fig. 5d-e). These results indicate that CD271 activation improves the outcome of topical treatment in cSCC.

### CD271 activation induces apoptosis, reduces invasion, and synergizes with the Trk inhibition effect in cSCC-derived spheroids

**Fig. 6:**
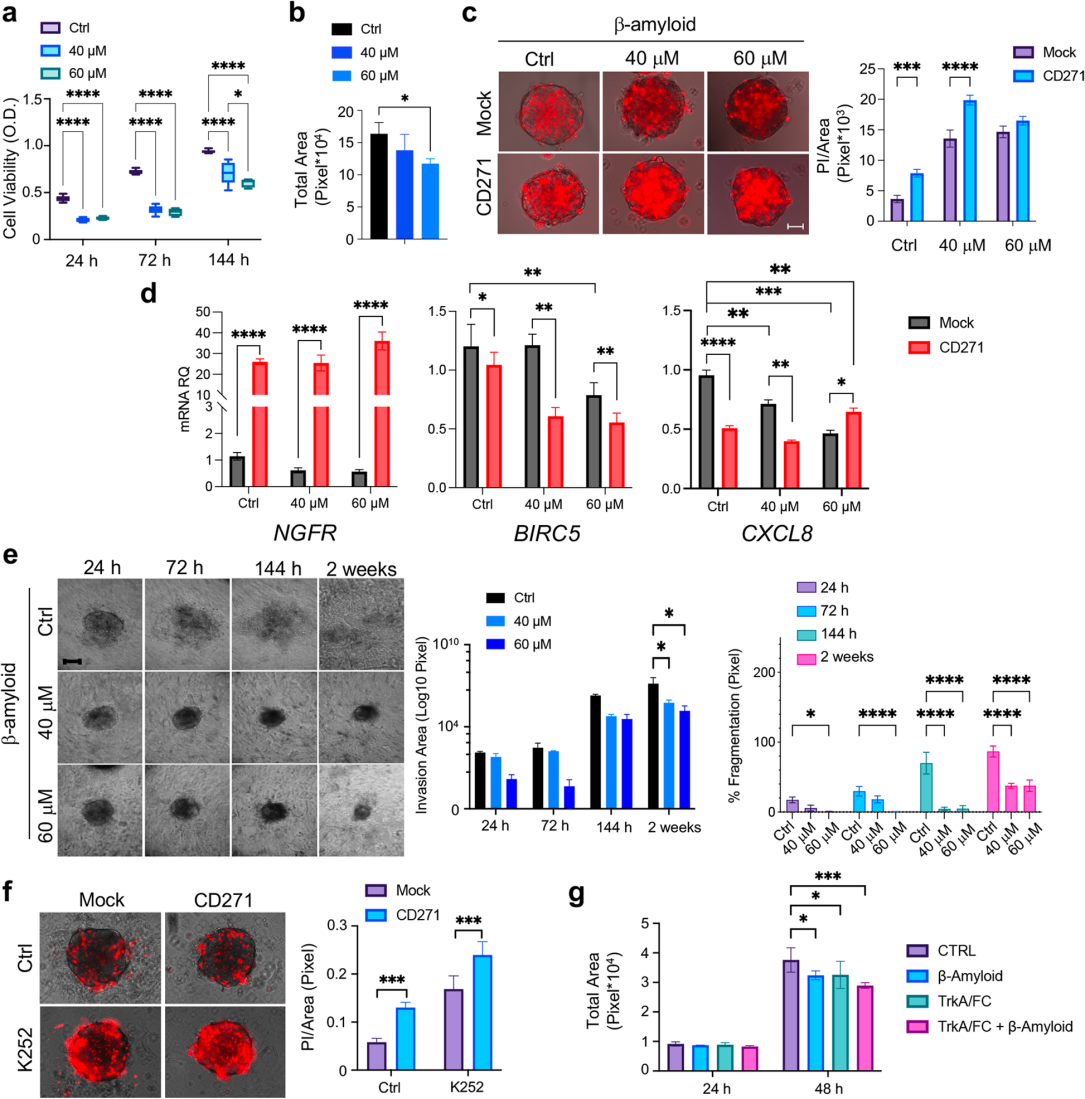
Effects of CD271 activation on cSCC spheroid viability and invasion **a** Cell viability (MTT assay) and **b** total area of SCC13 spheroids (n≥6) after β-Amyloid (40 μM and 60 μM) treatment as compared to control (PBS). **c** Left panel: Propidium Iodide (PI) staining (red)/brightfield on mock vs CD271-overexpressing SCC13 spheroids after β-Amyloid treatment as compared to control (PBS) at 48 h. Right: Bar chart representing PI-stained area (Pixel) (*n* = 3). Scale bar = 50 μm **d** *NGFR*, *BIRC5* and *CXCL8* mRNA levels (*n* = 8). **e** Left panel: Representative images of SCC13-derived implanted spheroid treated with β-Amyloid or PBS (control) and monitored for 2 weeks (scale bar = 50 μm). Middle panel: total invasion area. Right panel: percentage of fragmentation. **f** Left panel: PI staining/brightfield of CD271 and mock SCC13 spheroids treated with K252 (200 nM) or DMSO at 48 h. Right panel: Quantification of the PI staining/Total area ratio. Scale bar = 50 μm **g** Total area of SCC13 spheroids treated with TrkA/Fc (2 µg/ml), βAmyloid (40 μM) or the combinations TrkA/FC/βAmyloid (*n* = 3). Results are shown as mean ± SD of three independent experiments. Statistical analysis was performed using the two-way ANOVA and multiparametric T-test. *0.01 < *p* < 0.05, **0.001 < *p* < 0.01, ***0.0001 < *p* < 0.001, ****p < 0.0001

CD271 inhibits proliferation and stimulates differentiation in cSCC cells, pointing to a critical role of the receptor in blocking SCC development. To gain further insights into the mechanisms underlying CD271-dependent inhibition of proliferation in cSCC, we directly activated CD271 via its natural ligand β-Amyloid [46]. CD271 activation decreases cSCC cell viability and size of cSCC spheroids in a dose-dependent manner (Fig. 6a,b). Moreover, the amount of Propidium Iodide (PI) positive cells in SCC spheroids is increased by β-Amyloid treatment and exacerbated by combining β-Amyloid with CD271-overexpression (Fig. 6c). The same results were obtained in SCC12B spheroids (Fig. S6a-c). Furthermore, β-Amyloid treatment significantly reduces the expression of Survivin (*BIRC5*), which is further decreased by combination with CD271-overexpression. In addition, CD271 activation reduces the levels of CXCL8, associated with cancer progression, thus also interfering with SCC-derived tumor spheroid invasiveness (Fig. 6d).

To evaluate the impact of CD271 activation on spheroids invasion, SCC13 spheroids implanted in the collagen matrix I were further treated with β-Amyloid (Fig. 6e). The treatment reduces both spheroid total and invasion areas starting from 144 h up to 2 weeks. Moreover, a significantly lower % fragmentation was observed in spheroids treated with β-Amyloid, as compared to controls (Fig. 6e and Fig. S6d). Taken together, these results indicate that CD271 activation reduces the ability of cancer cells to disseminate within the tumor microenvironment and is potentially correlated with a lower metastatic potential.

Given the opposing effect of Trk receptors and CD271 in different cell settings [10, 47], we evaluated the effect of the combination treatment of CD271 activation and the alkaloid K252a, that specifically inhibits Trk phosphorylation, or the TrkA/Fc chimera, a soluble receptor that prevents binding of the NT to its receptor [48]. K252a significantly reduces the size of CD271-overexpressing spheroids to a higher degree as compared to mock spheroids (Fig. S6e). This was confirmed by the significant increment of apoptosis when CD271 is overexpressed and Trk signaling is inhibited (Fig. 6f). TrkA/Fc treatment is able to significantly reduce SCC13 spheroid growth and its effect synergizes with β-Amyloid treatment (Fig. 5g). These data reveal that modulating Trk and CD271 receptors results in an effective strategy for inhibiting cSCC cell viability.

### CD271 activation improves chemotherapy outcome and promotes macrophage recruitment in zebrafish

**Fig. 7:**
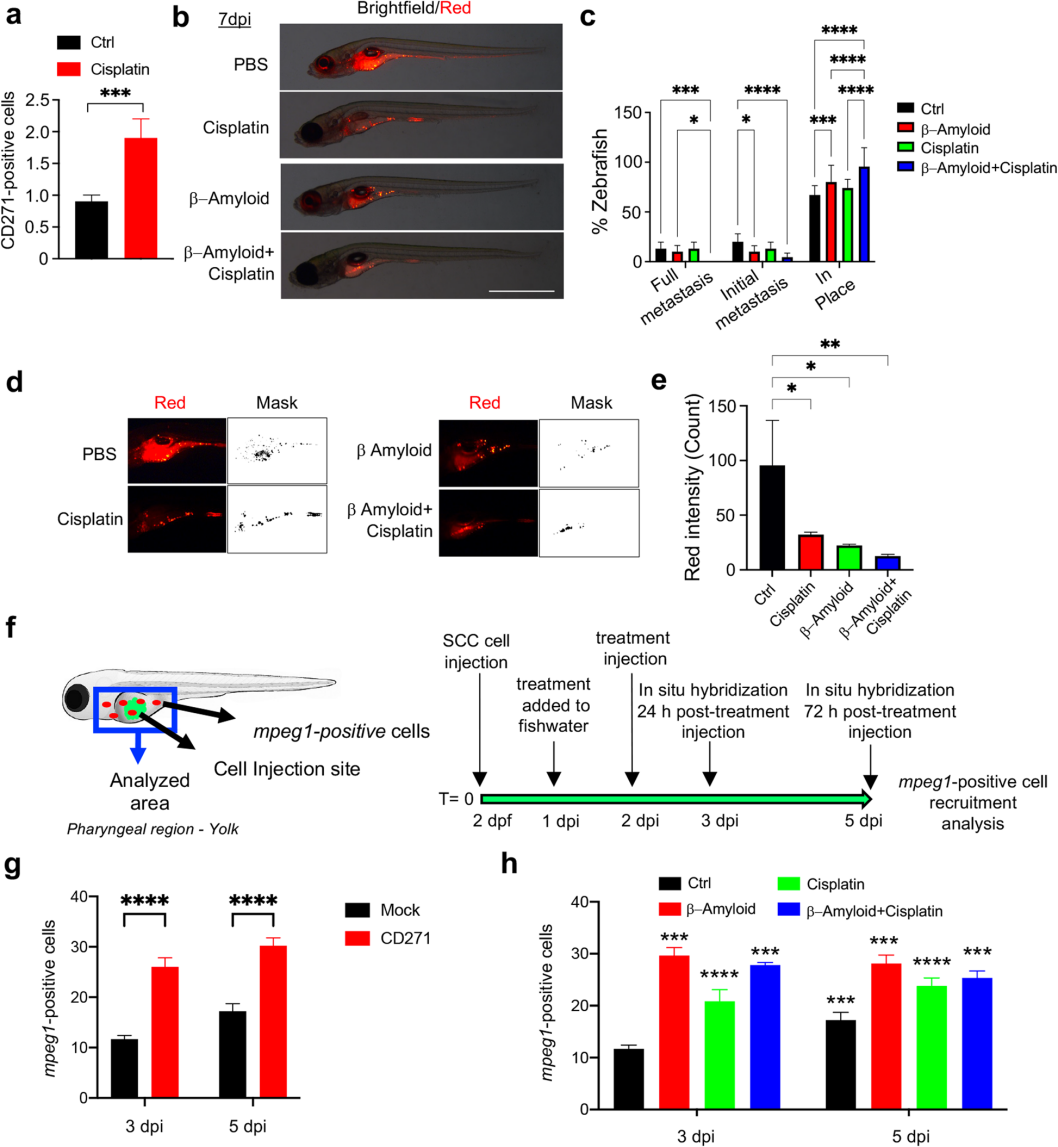
CD271 activation increases response to chemotherapy and promotes macrophage recruitment in zebrafish **a** CD271 expression in SCC13 cells treated with Cisplatin (5μg/mL) vs control by FACS. **b** Representative images of SCC13 cells (red) injected into zebrafish at 7dpi after treatments as described. Scale bar = 500µm. **c** cSCC metastasis quantified and classified as Full metastases, Initial metastases, or In place. **d-e** Quantification of the red fluorescence intensity of tumor mass in zebrafish by ImageJ software and ImageJ mask. **f** Left images: schematic representation of mpeg1 positive cells (red) in zebrafish injected with mock or CD271-overexpressing cells (green) within the analyzed area (blue). Right images: schematic representation of treatments. **g** Quantification of mpeg1-positive cells in zebrafish injected with mock or CD271-overexpressing cells and **h** in zebrafish injected with SCC13 cells after treatments as described, by whole-mount in situ hybridization. PBS was used as a control (*n* = 3, 3 independent evaluations). Results are shown as the mean percentage of three independent experiments (minimum of 20 injected zebrafish/condition). Statistical analysis was performed using the two-way ANOVA and multiparametric T-test. *0.01 < *p* < 0.05, **0.001 < *p* < 0.01, ***0.0001 < *p* < 0.001, ****p < 0.0001

Previous works have shown that several stimuli, such as proinflammatory or chemotherapeutic agents, as well as stress-responsive elements, lead to increased CD271 expression [22, 49–51]. Accordingly, SCC13 cells express higher amounts of CD271 when treated with cisplatin (Fig. 7a). Therefore, SCC13 cells were injected in zebrafish yolk and treated as reported in Fig. S7a. Specifically, cisplatin was added to fish water at 24 h from SCC13 cell injection, while β-Amyloid was injected at 2 dpi (day post-injection) (Fig. S7a). No significant differences in zebrafish viability are observed between treated animals and controls, suggesting that the treatment is not toxic at the concentration used (Fig. S7b). The combined treatment of cisplatin and β-Amyloid results in a marked reduction of initial metastases and the absence of full metastases, as compared to controls. In fact, more than 95% of cells remained in place. However, β-Amyloid was also effective in reducing metastases, by promoting up to 80% of zebrafish with in place cell conditions and a significant decrease of the initial metastasis (Fig. 7b-c and Fig. S7c). Moreover, a significant decrease in cell fluorescence intensity is observed in zebrafish treated with the combination of cisplatin and β-Amyloid (Fig. 7d-e).

A crosstalk between the nervous and the immune system has been previously described [52]. CD271 displays a pro-inflammatory role in chronic conditions [52, 53]. However, its function within the tumor microenvironment needs to be addressed. Macrophages represent the first line of defense in zebrafish larvae [54] and the analysis of the macrophage-specific marker *Macrophage-expressed gene (Mpeg)1* (*mpeg1.1* in zebrafish) [55] allows to explore the immune response in the zebrafish xenograft models. The *mpeg1.1* has been visualized by in situ hybridization [28] or by using the newly developed *mpeg1.1*:*GFP* and *mpeg1.1*:*mCherry* reporter fish [56]. Here, we used the *Tg(mpeg1:mCherry)g123* transgenic line and *mpeg1* in situ hybridization to analyze the macrophage recruitment following the injection of CD271-overexpressing cells or mock cells or with cisplatin and/or β-Amyloid treatment, as summarized in Fig. 7f. CD271 overexpression, cisplatin, β-Amyloid or combined treatment produced a significant increase of the mpeg1-positive cells at the site of injection when compared to controls (Fig. 7g-h and Fig. S8, S9). Interestingly, mpeg1-positive cell recruitment was higher at 3 dpi for cisplatin-β-Amyloid combined treatment compared to single treatments (Fig. 7h). These results suggest that CD271 exerts a protective effect against cSCC, by favoring innate immune response.

### CD271 expression correlates with innate immune cell recruitment in vivo and in vitro

**Fig. 8:**
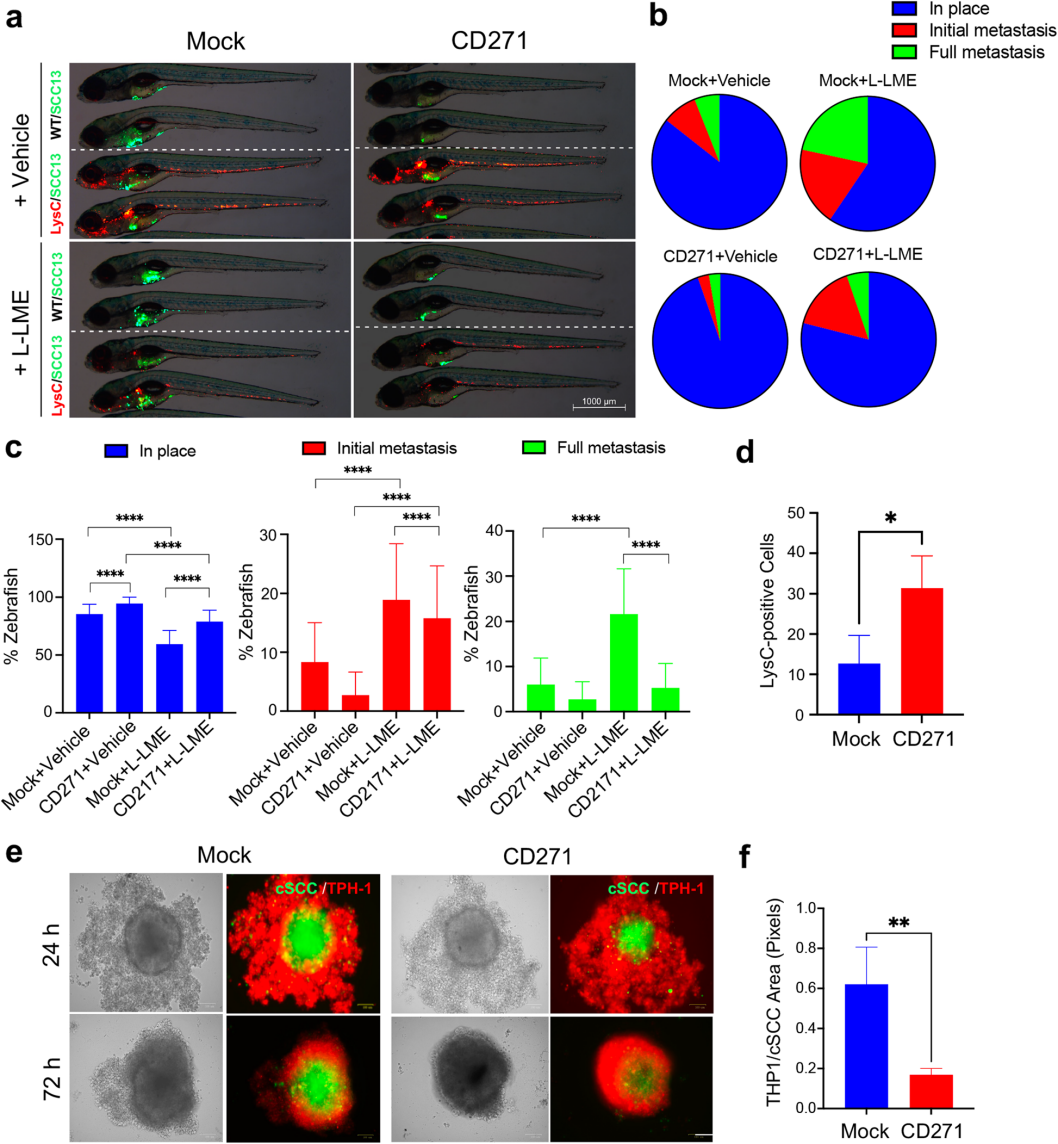
CD271 expression increases the immune cell recruitment in vivo and in vitro **a** Representative images of mock and CD271-overexpressing SCC13 cells (green) injected into WT (indicated as WT/SCC13 in the figure) or *Tg(lysC:DcRed)nz50* (indicated as LysC/SCC13) zebrafish with or without the simultaneous treatment with the L-LME. In the *Tg(lysC:DcRed2)nz50* zebrafish, leucocytes possess a red fluorescence. Scale bar = 500 µm. **b** and **c** cSCC metastases quantified and classified as Full metastases, Initial metastases, or In place. **d** Quantification of LysC-positive cells in zebrafish injected with mock or CD271-overexpressing cells **e** SCC13 cells were stained with Vybrant DIO Cell-Labeling Solution (green) and seeded in a 96-well plate coated with 1,5% of agar. cSCC spheroids were transduced by infection with mock or CD271 viral vectors. After 72 h, THP-1 cells were stained with Vybrant DII Cell-Labeling Solution (red) and co-cultured with cSCC spheroids. Pictures were made after 24 and 72 h from the THP-1 seeding. Scale bar = 100µm. **f** The invasion area of THP-1 was measured by ImageJ software. Results are shown as the mean percentage of three independent experiments (minimum of 20 injected zebrafish/condition). Statistical analysis was performed using the two-way ANOVA and multiparametric T-test. **0.001 < *p* < 0.01, ****p < 0.0001

To confirm our previous findings and define the involvement of CD271 in the recruitment of immune cells within the tumoral microenvironment, we performed mock or CD271-overexpressing cSCC cells injection into zebrafish larvae with or without immune cells ablation. Both Wild-Type (WT) or *Tg(lysC:DsRed2)nz50* zebrafish strain was used, and cSCC cells were injected with or without the simultaneous treatment with the L-Leucyl L-Leucine methyl esther compound (L-LME), a lysosomotropic compound known to be selectively toxic to macrophage, NK cells, cytotoxic T lymphocytes and skin mast cells [57, 58] (Fig. 8a). The immune cell ablation induced a significant increase in the percentage of zebrafish with initial and full metastases (Fig. 8b-c), suggesting that the zebrafish immune system is important in preventing cSCC cell metastasis. In particular, the block of cell spreading observed in zebrafish injected with CD271-overexpressing cells was partially reversed after leucocyte ablation with L-LME, suggesting that the expression of CD271 promotes the involvement of the immune cells in reducing the cSCC cell metastatic capacity (Fig. 8a-c). Furthermore, confirming our previous result (Fig. 7g, h), the number of LysC-positive cells within the tumor area was increased by injecting CD271-overexpressing cells (Fig. 8d).

To introduce human myeloid immune cells in our cSCC models, we set up several 2D/3D co-culture assays to determine the ability of cSCC to induce monocyte infiltration or invasion according to the expression level of CD271 (Fig. 8 and Fig. S10). THP-1 cells, a human monocyte cell line [59], were used for these purposes. We found that THP-1 cells increase their infiltrating capacity in Matrigel when stimulated by CD271-derived supernatants or co-cultured with CD271-overexpressing cSCC cells. This effect was significantly exacerbated when THP-1 cells were cultured with CD271-overexpressing cells and stimulated with CD271 conditional media (Fig. S10a-b). Moreover, THP-1 cells increased their capacity to migrate in a scratch assay wound when cultured in the presence of CD271-derived supernatant as compared to mock. Interestingly, THP-1 cells differentiated into macrophages by TPA (Fig. S11a) and treated with CD271-derived supernatants showed a modification in their morphology, from a round shape to a more mesenchymal one (Fig. S11b). Finally, we set up a new 3D spheroidal model, where mock and CD271-overexpressing cSCC spheroids were co-cultured with THP-1 cells. Surprisingly, we found a significant decrease in THP-1 infiltrating area/cSCC spheroid area ratio in CD271-overexpressing spheroids as compared to mock, which means that CD271-overexpressing cells can recruit THP-1 cells more efficacy than mock cells (Fig. 8e-f). All these results confirmed that CD271 exerts a role in the recruitment of immune cells in the tumor microenvironment, which could have a role in inhibiting tumor aggressiveness. However, more studies are necessary to understand the precise role of CD271 in immune cell recruitment and macrophage polarization.

## Discussion

In recent years, significant progress has been made in dissecting the molecular mechanisms underlying cSCC development. In particular, loss of differentiation is widely accepted as a critical event, becoming the point of no return toward cSCC progression [60]. In this context, the NT network has a key function in modulating the physiological balance between proliferation and differentiation of the epidermal cells and a role for CD271 in early keratinocyte differentiation has been reported [11, 12]. Nevertheless, the role of CD271 in cSCC remains to be defined. CD271/proBDNF signaling mediates apoptosis in basal cell carcinoma [61], thus possibly preventing cancer proliferation and development. On the other hand, CD271 has been proposed as a marker of tumor-initiating-cells in oral human and murine SCC cells, and CD271 activation by NGF results in a more invasive phenotype [62], most likely involving Trk-CD271 heterodimer signaling. Because CD271 plays a critical role in early differentiation from KSC [11], where the cutaneous SCC originates [63], we focused on determining its functional role in cSCC. Herein, we present unprecedented data showing that CD271 plays a protective role in cSCC, by preventing progression and invasion.

While the precise role of CD271 in the early events related to the origin of cSCC remains to be elucidated, the receptor might be involved in the molecular alterations leading to cSCC progression. In fact, CD271 is detectable in WD cSCC tumors, as compared to the more aggressive MD/PD cSCCs. Independently of the tumor grade, the exogenous expression of CD271 is able to reduce cSCC cell proliferation and the level of several markers associated with an aggressive phenotype in patient-derived cSCC spheroids, by increasing differentiation. Similarly, CD271 overexpression in the WD SCC cell line-derived spheroids reduces invasiveness, while its silencing increases viability, thus validating the data obtained with the primary tumors. The prognostic classification for primary cSCC, initially based on the TNM system of UICC (Union for International Cancer Control) and AJCC (American Joint Committee on Cancer), and further revisited [3], indicates the poorly differentiated histopathological characteristics as determinants for the risk of recurrence and distant metastases. Therefore, the activation of CD271-dependent activities might result in a key factor that could avoid the rearrangement of the neoplastic signature in favor of a high-risk lesion, ameliorating the efficacy of the curative intervention.

Furthermore, by combining H&E staining with the recently developed physical cytometry [26], we were able to demonstrate for the first time the deep structural organization of the tumor mass. CD271-overexpressing spheroids are more compact and smaller in size and weight as compared to controls. They also display a greater density, which is accounted for by the low cell number with respect to extracellular components, suggesting that CD271 could regulate the keratinization of cSCC cells, as indicated by the RNA-sequencing, and in agreement with its prodifferentiative role in healthy skin [11].

Topical treatments, such as PDT, 5-FU, or Imiquimod [64–66] are often taken into consideration in cSCC precursor lesions such as AK, in cSCC in situ, or in elderly or unhealthy patients when surgery could be risky. Moreover, this is an option for tumors in cosmetically sensitive areas where surgery may cause a disfiguring scar. CD271 overexpression significantly reduces cell viability induced in tumor spheroids by PDT or chemotherapy, indicating that CD271 expression improves the outcome of PDT and 5-FU in vitro and in vivo.

It is of great clinical relevance that activating the CD271 receptor in cancer cells by a ligand could potentially open the field to new therapeutic strategies for cSCC. β-Amyloid, the natural CD271 ligand, administered as peptide correspondent to 25–35 residues of the full protein, reduces the size and the invasion area of spheroids while increasing apoptosis induced by CD271 overexpression. Therefore, by using a commercially available peptide, we provide evidence of an anti-tumoral effect produced by the activation of CD271 through a small molecule. This result could represent the starting point for novel pharmacological development targeting the neurotrophin receptor without blocking its own signaling, as recently discussed for other types of epithelial or non-epithelial cancer [67, 68].

While zebrafish is being widely used in cancer [44], we have first adapted the model for cSCC. Injection of CD271-overexpressing cSCC cells determines a marked reduction of metastases in zebrafish larvae, confirmed by the decrease in the expression of proliferative and invasiveness markers. The same effect was obtained by the addition of β-Amyloid or cisplatin, which upregulates CD271 expression, and by their combination. These data clearly show that CD271 expression or activation can revert the aggressive phenotype in cSCC. The increased CD271 expression due to chemotherapeutics, where the induction of CD271 mostly occurred because of p53 activation [69] or anti-inflammatory drugs [70] has recently been evaluated. Therefore, our data indicate that the specific activation of CD271 within the context of a clinical protocol involving the use of platinum-based or 5-FU therapeutics [71, 72] could determine a higher sensitivity of cSCC cancer cells to differentiation and cell death, especially for the advanced conditions.

Given the clinical development of a new class of compounds targeting the NTRK (Neurotrophic Tyrosine Kinase receptor) fusion protein [14, 15], we evaluated the effect in the combination treatment of CD271 activation and Trk inhibition. Both the alkaloid K252a, which specifically inhibits Trk-receptor signaling [48] and the addition of TrkA/Fc chimera resulted in decreased viability, confirming the dependence of cSCC cells from the autocrine NTs, as also demonstrated in other types of cancers [73].

Little is known about the correlation between CD271 and the immune system in the context of cancer. Tumor metastatic behavior is modified by immune cells that, in the early stages of tumorigenesis, present neoantigens to CD8 + T cells to destroy the tumor [74]. To define the role of CD271 in the immune response against cSCC, we took advantage of the well-known model of innate immunity involving macrophages in zebrafish [28]. We show the essential role that CD271 plays in the innate immune response against cSCC in vivo, by recruiting mpeg1-positive cells. Moreover, the increasing amount of initial metastasis in leucocyte-depleted zebrafish, injected with CD271-overexpressing cells, sustains a potential role for CD271-dependent macrophage activation in promoting a significative reduction of the cSCC tumor mass. Furthermore, by using both 2D and 3D human models of cSCC and monocyte cell interplay, we provide evidence of CD271-related immune cell differentiation and activation in response to cancer cell signals. However, the function of CD271 in relation to the immune microenvironment in cSCC needs further investigation, this finding strongly suggests the tumor suppressor activity of the receptor.

## Conclusion

In conclusion, our findings strongly support a tumor suppression function for CD271 in cSCC, through the inhibition of cancer cell viability and dissemination. This activity could be exploited alone, in combination with the available topical treatments or with the recently developed Trk inhibitors, to obtain a more effective clinical response in cSCC.

## Supplementary Information

**Additional file 1.**

## Data Availability

RNAseq data have been deposited in the Gene Expression Omnibus (GEO) site with the accession number GSE178845.

## Abbreviations

cSCC: Cutaneous squamous cell carcinoma
WD: Well-differentiated
MD/PD: Moderately/Poorly Differentiated
PTD: Photodynamic therapy
AK: Actinic keratosis
NTs: Neurotrophins
NTRK: Neurotrophic Tyrosine Kinase receptor
KRT1: Keratin 1
KRT13: Keratin 13
KRT10: Keratin 10
EMT: Epithelial to Mesenchymal transition
5FU: 5-Fluorouracil
PI: Propidium Iodide

## References

[1] Nagarajan P, Asgari MM, Green AC, Guhan SM, Arron ST, Proby CM (2019). Keratinocyte carcinomas: current concepts and future research priorities. Clin Cancer Res.

[2] Leiter U, Keim U, Garbe C. Epidemiology of Skin Cancer: Update 2019. In: Reichrath J, editor. Sunlight, Vitamin D and Skin Cancer. Cham: Springer International Publishing; 2020 [cited 2022 Jan 27]. p. 123–39. (Advances in Experimental Medicine and Biology; vol. 1268). Available from: http://link.springer.com/10.1007/978-3-030-46227-7_6 10.1007/978-3-030-46227-7_6 32918216

[3] Stratigos AJ, Garbe C, Dessinioti C, Lebbe C, Bataille V, Bastholt L (2020). European interdisciplinary guideline on invasive squamous cell carcinoma of the skin: Part 2 Treatment. Eur J Cancer.

[4] Farasat S, Yu SS, Neel VA, Nehal KS, Lardaro T, Mihm MC (2011). A new American Joint Committee on Cancer staging system for cutaneous squamous cell carcinoma: creation and rationale for inclusion of tumor (T) characteristics. J Am Acad Dermatol.

[5] Thompson AK, Kelley BF, Prokop LJ, Murad MH, Baum CL (2016). Risk factors for cutaneous squamous cell carcinoma recurrence, metastasis, and disease-specific death: a systematic review and meta-analysis. JAMA Dermatol.

[6] Fania L, Didona D, Di Pietro FR, Verkhovskaia S, Morese R, Paolino G (2021). Cutaneous squamous cell carcinoma: from pathophysiology to novel therapeutic approaches. Biomedicines.

[7] Lobl MB, Clarey DD, Higgins S, Sutton A, Wysong A (2022). Sequencing of cutaneous squamous cell carcinoma primary tumors and patient-matched metastases reveals ALK as a potential driver in metastases and low mutational concordance in immunocompromised patients. JID Innov Skin Sci Mol Popul Health.

[8] Bothwell M. Recent advances in understanding context-dependent mechanisms controlling neurotrophin signaling and function. F1000Research. 2019;8:F1000 Faculty Rev-1658. 10.12688/f1000research.19174.1 PMC6758832 31583078

[9] Truzzi F, Marconi A, Atzei P, Panza MC, Lotti R, Dallaglio K (2011). p75 neurotrophin receptor mediates apoptosis in transit-amplifying cells and its overexpression restores cell death in psoriatic keratinocytes. Cell Death Differ.

[10] Pincelli C, Marconi A (2010). Keratinocyte stem cells: friends and foes. J Cell Physiol.

[11] Truzzi F, Saltari A, Palazzo E, Lotti R, Petrachi T, Dallaglio K (2015). CD271 mediates stem cells to early progeny transition in human epidermis. J Invest Dermatol.

[12] Lotti R, Palazzo E, Quadri M, Dumas M, Schnebert S, Biondini D (2022). Isolation of an “early” transit amplifying keratinocyte population in human epidermis: a role for the low affinity neurotrophin receptor CD271. Stem Cells.

[13] Khotskaya YB, Holla VR, Farago AF, Mills Shaw KR, Meric-Bernstam F, Hong DS (2017). Targeting TRK family proteins in cancer. Pharmacol Ther.

[14] Doebele RC, Davis LE, Vaishnavi A, Le AT, Estrada-Bernal A, Keysar S (2015). An oncogenic NTRK fusion in a patient with soft-tissue sarcoma with response to the tropomyosin-related kinase inhibitor LOXO-101. Cancer Discov.

[15] Rolfo C, Ruiz R, Giovannetti E, Gil-Bazo I, Russo A, Passiglia F (2015). Entrectinib: a potent new TRK, ROS1, and ALK inhibitor. Expert Opin Investig Drugs.

[16] Foerster Y, Stöver T, Wagenblast J, Diensthuber M, Balster S, Gabrielpillai J (2019). Relevance of neurotrophin receptors CD271 and TrkC for prognosis, migration, and proliferation in head and neck squamous cell carcinoma. Cells.

[17] Palazzo E, Marconi A, Pincelli C, Morasso MI (2019). Do DLX3 and CD271 protect human keratinocytes from squamous tumor development?. Int J Mol Sci.

[18] Dallaglio K, Petrachi T, Marconi A, Truzzi F, Lotti R, Saltari A (2014). Expression of nuclear survivin in normal skin and squamous cell carcinoma: a possible role in tumour invasion. Br J Cancer.

[19] Okumura T, Shimada Y, Imamura M, Yasumoto S (2003). Neurotrophin receptor p75(NTR) characterizes human esophageal keratinocyte stem cells in vitro. Oncogene.

[20] Kiyosue T, Kawano S, Matsubara R, Goto Y, Hirano M, Jinno T (2013). Immunohistochemical location of the p75 neurotrophin receptor (p75NTR) in oral leukoplakia and oral squamous cell carcinoma. Int J Clin Oncol.

[21] Verbeke S, Meignan S, Lagadec C, Germain E, Hondermarck H, Adriaenssens E (2010). Overexpression of p75(NTR) increases survival of breast cancer cells through p21(waf1). Cell Signal.

[22] Khwaja FS, Quann EJ, Pattabiraman N, Wynne S, Djakiew D (2008). Carprofen induction of p75NTR-dependent apoptosis via the p38 mitogen-activated protein kinase pathway in prostate cancer cells. Mol Cancer Ther.

[23] Ratliff TL (2005). Ibuprofen inhibits survival of bladder cancer cells by induced expression of the p75NTR tumor suppressor protein. J Urol.

[24] Quadri M, Comitato A, Palazzo E, Tiso N, Rentsch A, Pellacani G (2022). Activation of cGMP-dependent protein kinase restricts melanoma growth and invasion by interfering with the EGF/EGFR pathway. J Invest Dermatol.

[25] Saltari A, Truzzi F, Quadri M, Lotti R, Palazzo E, Grisendi G (2016). CD271 down-regulation promotes melanoma progression and invasion in three-dimensional models and in zebrafish. J Invest Dermatol.

[26] Cristaldi DA, Sargenti A, Bonetti S, Musmeci F, Delprete C, Bacchi F (2020). A reliable flow-based method for the accurate measure of mass density, size and weight of Live 3D tumor spheroids. Micromachines.

[27] Quadri M, Marconi A, Sandhu SK, Kiss A, Efimova T, Palazzo E (2022). Investigating cutaneous squamous cell carcinoma in vitro and in vivo: novel 3D tools and animal models. Front Med.

[28] Thisse C, Thisse B, Schilling TF, Postlethwait JH (1993). Structure of the zebrafish snail1 gene and its expression in wild-type, spadetail and no tail mutant embryos. Dev Camb Engl.

[29] Codolo G, Facchinello N, Papa N, Bertocco A, Coletta S, Benna C (2022). Macrophage-mediated melanoma reduction after HP-NAP treatment in a zebrafish xenograft model. Int J Mol Sci.

[30] Chen JJ, Harris JP, Kong CS, Sunwoo JB, Divi V, Horst KC (2018). Clinical perineural invasion of cutaneous head and neck cancer: Impact of radiotherapy, imaging, and nerve growth factor receptors on symptom control and prognosis. Oral Oncol.

[31] Lambert SR, Mladkova N, Gulati A, Hamoudi R, Purdie K, Cerio R (2014). Key differences identified between actinic keratosis and cutaneous squamous cell carcinoma by transcriptome profiling. Br J Cancer.

[32] Moll R, Moll I, Franke WW (1984). Differences of expression of cytokeratin polypeptides in various epithelial skin tumors. Arch Dermatol Res.

[33] Fu M, Wang C, Li Z, Sakamaki T, Pestell RG (2004). Minireview: Cyclin D1: normal and abnormal functions. Endocrinology.

[34] Bajpai D, Mehdizadeh S, Uchiyama A, Inoue Y, Sawaya A, Overmiller A (2021). Loss of DLX3 tumor suppressive function promotes progression of SCC through EGFR–ERBB2 pathway. Oncogene.

[35] Darwiche N, Ryscavage A, Perez-Lorenzo R, Wright L, Bae DS, Hennings H (2007). Expression profile of skin papillomas with high cancer risk displays a unique genetic signature that clusters with squamous cell carcinomas and predicts risk for malignant conversion. Oncogene.

[36] Kalluri R, Weinberg RA (2009). The basics of epithelial-mesenchymal transition. J Clin Invest.

[37] Xie S, Xu H, Shan X, Liu B, Wang K, Cai Z (2015). Clinicopathological and prognostic significance of survivin expression in patients with oral squamous cell carcinoma: evidence from a meta-analysis. PLoS ONE.

[38] Alassaf E, Mueller A (2020). The role of PKC in CXCL8 and CXCL10 directed prostate, breast and leukemic cancer cell migration. Eur J Pharmacol.

[39] Massa SM, Xie Y, Yang T, Harrington AW, Kim ML, Yoon SO (2006). Small, nonpeptide p75NTR ligands induce survival signaling and inhibit proNGF-induced death. J Neurosci Off J Soc Neurosci.

[40] Casas E, Kim J, Bendesky A, Ohno-Machado L, Wolfe CJ, Yang J (2011). Snail2 is an essential mediator of Twist1-induced epithelial mesenchymal transition and metastasis. Cancer Res.

[41] Hou YY, Cao WW, Li L, Li SP, Liu T, Wan HY (2011). MicroRNA-519d targets MKi67 and suppresses cell growth in the hepatocellular carcinoma cell line QGY-7703. Cancer Lett.

[42] Sahai E (2007). Illuminating the metastatic process. Nat Rev Cancer.

[43] Lloyd C, Yu QC, Cheng J, Turksen K, Degenstein L, Hutton E (1995). The basal keratin network of stratified squamous epithelia: defining K15 function in the absence of K14. J Cell Biol.

[44] Bootorabi F, Manouchehri H, Changizi R, Barker H, Palazzo E, Saltari A (2017). Zebrafish as a model organism for the development of drugs for skin cancer. Int J Mol Sci.

[45] Zwiebel S, Baron E (2011). PDT in squamous cell carcinoma of the skin. G Ital Dermatol E Venereol Organo Uff Soc Ital Dermatol E Sifilogr.

[46] Yaar M, Zhai S, Pilch PF, Doyle SM, Eisenhauer PB, Fine RE (1997). Binding of beta-Amyloid to the p75 neurotrophin receptor induces apoptosis. A possible mechanism for Alzheimer’s disease. J Clin Invest.

[47] Reichardt LF (2006). Neurotrophin-regulated signalling pathways. Philos Trans R Soc Lond B Biol Sci.

[48] Palazzo E, Marconi A, Truzzi F, Dallaglio K, Petrachi T, Humbert P (2012). Role of neurotrophins on dermal fibroblast survival and differentiation. J Cell Physiol.

[49] White DE, Burchill SA (2010). Fenretinide-dependent upregulation of death receptors through ASK1 and p38α enhances death receptor ligand-induced cell death in Ewing’s sarcoma family of tumours. Br J Cancer.

[50] Gravina GL, Marampon F, Sanità P, Mancini A, Colapietro A, Scarsella L (2016). Increased expression and activity of p75NTR are crucial events in azacitidine-induced cell death in prostate cancer. Oncol Rep.

[51] Gao W, Xu S, Zhang M, Liu S, Siu SPK, Peng H (2020). NADPH oxidase 5α promotes the formation of CD271 tumor-initiating cells in oral cancer. Am J Cancer Res.

[52] Minnone G, De Benedetti F, Bracci-Laudiero L (2017). NGF and its receptors in the regulation of inflammatory response. Int J Mol Sci.

[53] Jacobs BL, Smaldone MC, Tyagi V, Philips BJ, Jackman SV, Leng WW (2010). Increased nerve growth factor in neurogenic overactive bladder and interstitial cystitis patients. Can J Urol.

[54] van der Sar AM, Appelmelk BJ, Vandenbroucke-Grauls CMJE, Bitter W (2004). A star with stripes: zebrafish as an infection model. Trends Microbiol.

[55] Ellett F, Pase L, Hayman JW, Andrianopoulos A, Lieschke GJ (2011). mpeg1 promoter transgenes direct macrophage-lineage expression in zebrafish. Blood.

[56] Ferrero G, Gomez E, Lyer S, Rovira M, Miserocchi M, Langenau DM (2020). The macrophage-expressed gene (mpeg) 1 identifies a subpopulation of B cells in the adult zebrafish. J Leukoc Biol.

[57] Thiele DL, Lipsky PE. The immunosuppressive activity of L-leucyl-L-leucine methyl ester: selective ablation of cytotoxic lymphocytes and monocytes. J Immunol Baltim Md 1950. 1986;136(3):1038–48. 2934477

[58] Hagforsen E, Paivandy A, Lampinen M, Weström S, Calounova G, Melo FR (2015). Ablation of human skin mast cells in situ by lysosomotropic agents. Exp Dermatol.

[59] Chanput W, Mes JJ, Wichers HJ (2014). THP-1 cell line: An in vitro cell model for immune modulation approach. Int Immunopharmacol.

[60] Schmults CD, Karia PS, Carter JB, Han J, Qureshi AA (2013). Factors predictive of recurrence and death from cutaneous squamous cell carcinoma: a 10-year, single-institution cohort study. JAMA Dermatol.

[61] Lu Q, Qu Y, Ding Y, Kang X (2021). p75NTR/proBDNF modulates basal cell carcinoma (BCC) immune microenvironment via necroptosis signaling pathway. J Immunol Res.

[62] Chung MK, Jung YH, Lee JK, Cho SY, Murillo-Sauca O, Uppaluri R (2018). CD271 Confers an Invasive and Metastatic Phenotype of Head and Neck Squamous Cell Carcinoma through the Upregulation of Slug. Clin Cancer Res Off J Am Assoc Cancer Res.

[63] Shukla S, Meeran SM (2014). Epigenetics of cancer stem cells: pathways and therapeutics. Biochim Biophys Acta.

[64] Trodello C, Pepper JP, Wong M, Wysong A (2017). Cisplatin and cetuximab treatment for metastatic cutaneous squamous cell carcinoma: a systematic review. Dermatol Surg Off Publ Am Soc Dermatol Surg Al.

[65] Viros A, Hayward R, Martin M, Yashar S, Yu CC, Sanchez-Laorden B (2013). Topical 5-fluorouracil elicits regressions of BRAF inhibitor-induced cutaneous squamous cell carcinoma. J Invest Dermatol.

[66] Austin E, Jagdeo J. An in vitro approach to photodynamic therapy. J Vis Exp. 2018;(138):58190. 10.3791/58190. 10.3791/58190 PMC6128114 30176015

[67] Morita S, Mochizuki M, Wada K, Shibuya R, Nakamura M, Yamaguchi K (2019). Humanized anti-CD271 monoclonal antibody exerts an anti-tumor effect by depleting cancer stem cells. Cancer Lett.

[68] El Touny LH, Henderson F, Djakiew D (2010). Biochanin A reduces drug-induced p75NTR expression and enhances cell survival: a new in vitro assay for Screening Inhibitors of p75NTR expression. Rejuvenation Res.

[69] Redmer T, Walz I, Klinger B, Khouja S, Welte Y, Schäfer R (2017). The role of the cancer stem cell marker CD271 in DNA damage response and drug resistance of melanoma cells. Oncogenesis.

[70] Khwaja F, Allen J, Lynch J, Andrews P, Djakiew D (2004). Ibuprofen inhibits survival of bladder cancer cells by induced expression of the p75NTR tumor suppressor protein. Cancer Res.

[71] Fu T, Aasi SZ, Hollmig ST (2016). Management of high-risk squamous cell carcinoma of the skin. Curr Treat Options Oncol.

[72] Forastiere AA, Metch B, Schuller DE, Ensley JF, Hutchins LF, Triozzi P (1992). Randomized comparison of cisplatin plus fluorouracil and carboplatin plus fluorouracil versus methotrexate in advanced squamous-cell carcinoma of the head and neck: a Southwest Oncology Group study. J Clin Oncol.

[73] Regua AT, Doheny D, Arrigo A, Lo HW (2019). Trk receptor tyrosine kinases in metastasis and cancer therapy. Discov Med.

[74] Güç E, Pollard JW (2021). Redefining macrophage and neutrophil biology in the metastatic cascade. Immunity.

