# Supplementary material for: CD271 activation prevents low to high-risk progression of cutaneous squamous cell carcinoma and improves therapy outcomes
Source: J Exp Clin Cancer Res. 2023 Jul 13;42:167. doi: 10.1186/s13046-023-02737-7 (PMC10339543; doi:10.1186/s13046-023-02737-7)
Supplement: Supplementary file 1 — Additional file 1. [file 13046_2023_2737_MOESM1_ESM.docx]

**Supplementary Materials**

**Materials and methods**

**Clinical Sample recruitment and biopsy processing**

cSCC biopsies were provided by the Dermatological Clinic of the hospital of Modena and the diagnosis was confirmed by the pathologist of the Department of Diagnostic, Clinic and Public Health Medicine of the University of Modena and Reggio Emilia. The use of cSCC biopsies was approved by the Ethical Committee of Area Vasta Emilia Nord (Protocol n°184/10 and 353/2017). After diagnoses, cSCC biopsies were immersed in a solution of DMEM (Dulbecco’s Modified Eagle Medium; Lonza, Basel, Switzerland) and 1% of Penicillin/Streptomycin/Amphotericin (PSA – (Sigma-Aldrich, Missouri, USA) and incubated for 2 hours at 4°C. Subsequently, the biopsies were incubated in DMEM added with Dispase (200 U/ml- Roche, Basel, Switzerland), Collagenase I (200 U/ml – Roche, Basel, Switzerland) and DNase I (70 U/ml – Roche, Basel, Switzerland) for 2 hours at 37°C in agitation. Patients-derived cells were maintained on a 3T3 feeder-layer in DMEM/HAM’SF12 complete medium as described in Palazzo et al. 2019 and then seeded on a collagen IV coated plate in KGM medium (Lonza, Basel, Switzerland).

**Cell or SCC spheroid chemical treatments**

SCC12B, SCC13, and SCC15 cell lines were purchased from ATCC (Teddington, UK) and maintained in SCC medium (45% DMEM (Dulbecco’s Modified Eagle Medium; Lonza, Basel, Switzerland), 45% Ham’s F12 (Lonza, Basel, Switzerland), 10% Fetal Bovine Serum, 2% L-Glutamine, 1% Penicillin/Streptomycin/Amphotericin, 1M HEPES, and 400 ng/mL Hydrocortisone (Sigma-Aldrich, Missouri, USA), as previously described (Palazzo et al., 2019).

Patients-derived cells were maintained in DMEM/HAM’SF12 containing insulin (5 μg/ml, transferrin (5 μg/ml), triiodothyronine (2 nM), hydrocortisone (0.4 μg/ml), adenine (180 nM) mouse epidermal growth factor (10 nm/ml) (Sigma-Aldrich, Missouri, USA) and 10% FBS on 3T3 feeder-layer for some passage, and then seeded on collagen IV coated plate in KGM medium (Lonza, Basel, Switzerland)

SCC spheroid formation was obtained by the liquid overlay method, as previously indicated (Palazzo et al., 2019).

According to experimental requirements, spheroids were treated with βAmyloid (40 or 60 μM, GenScript, Genewiz, Germany), TrkA inhibitor K252 (200 nM, Sigma-Aldrich, St. Louis, Missouri, USA), Cisplatin (5 μg/ml, Pfizer, New York, USA), 5 Fluor-Uracil (1, 10 or 50 μg/ml, Accord Healthcare Limited, Middlesex, UK) and TrkA/FC chimera (2 μg/ml, R&D Systems, Minneapolis, USA).

Photodynamic therapy (PDT**)** was performed on SCC13 spheroids by modifying Austin and Jagdeo 2018 protocol (Austin et al., 2018). Briefly, CD271-overexpressing and mock spheroids, as well as untreated SCC13 spheroids, were added with aminolevunic acid (Metvix ®, 1:10 dilution) for 15 minutes, 1 or 3 hours before exposure to Aktilite CL128 lamp. Immediately after exposure, CD271-overexpressing and mock spheroids underwent media change.

**Cell transfection or infection**

For CD271 downregulation, SCC13 spheroids were maintained 24 hours in an antibiotic-free medium and then transfected with CD271 or scrambled siRNA (Dharmacon Inc, Lafayette, CO, USA). 72 hours after transfection, cells or spheroids were monitored or collected for subsequent experiments.

Patient-derived cSCC spheroids and SCC cell lines spheroids were transduced by infection with viral supernatant generated by CD271-LNSN packaging cells or by empty vector AM12 packaging cells, in the presence of polybrene (8 μg/ml, Sigma-Aldrich, St. Louis, Missouri, USA). 72 hours after infection cells or spheroids were monitored or collected for subsequent experiments.

**MTT Assay**

5×10^3^ cells from cSCC lines or patient-derived were seeded in 96-well culture plates to form spheroids. 72 hours after transfection or transduction or 24 and 48 hours after specific treatment, spheroids were incubated with 0.5% MTT (3-(4,5-dimethylthiazol-2-yl)-2,5-diphenyltetrazolium bromide, Sigma-Aldrich). MTT was added to spheroids and incubated for 4 hours at 37°C. Subsequently, formazan dye crystals were dissolved with 100 µl of Isopropanol with 0.04 N HCl. The MTT reaction was evaluated at 540 nm with a reference filter of 650 nm.

**Measurement of weight, diameter and mass density of spheroids with W8™**

Mock or CD271 SCC13 spheroids were analyzed with W8™ by CellDynamics (Bologna, Italy). The adopted method is based on tracking the sample’s motion, when free-falling into a vertical flow-channel while the flow is at rest, to calculate its terminal velocity. This is achieved by using customized software that relies on a specific physical method, combined with detailed statistical analyses, as previously indicated (Cristaldi et al., 2020). Briefly, the device is composed of a fluidic-core chip, equipped with a bright-field imaging setup, a peristaltic pump, a temperature sensor and a flow-circuit for the introduction and elimination of samples. Furthermore, the software assigns a circular reference to each image of the falling sample, allowing the extrapolation of the final radius from the physical calculation, as the average of the maximum radii obtained from each repetition. The terminal velocity is whereas calculated from the vertical position of the falling sample, and for each repetition, a linear regression plot is derived. During the entire analysis, different internal data control is performed: (i) initial visual screening of the operator; (ii) circular reference assignment; (iii) measurements repetition; (iv) calculation of the average radius; (v) linear regression plot analysis; and (vi) statistical validation with outlier’s elimination. All these checkpoints ensure the conformity of the analyzed samples in term of shape and size. A minimum of 10 single spheroids was analyzed for every test condition and values were extrapolated from at least 10 repetitions.

**Cutaneous SCC Skin equivalent**

SCC reconstructs were obtained by seeding SCC13 cell lines on dermal equivalents, generated by human primary dermal fibrobl­­­­asts induced type I collagen contraction, as previously reported (Saltari et al., 2016). Briefly, for dermal reconstructs, 0.5 ml of a cell free collagen solution (1.35 mg/ml rat tail type I collagen in DMEM with 10% FBS and 1% Pen/Strep) was added to tissue culture inserts (Transwell, Costar, Cambridge, MA) in 12-well plates. This precoated layer was overlaid with 1 ml of fibroblasts mixed with collagen type I solution (15x10^4^/ml). After 4 days of incubation at 37°C, 25x10^4^ of SCC13 mock or CD271 cells were seeded on dermal reconstructs, incubated for 1 hour at 37°C and then submerged in SCC medium for 4 days. Finally, skin reconstructs were exposed to the air and medium was changed every two days. After 12-day, skin reconstructs were fixed with formalin for 2 hours at RT, dehydrated and embedded in paraffin for subsequent analysis.

**Collagen I invasion assay**

SCC13 cell lines were seeded on pre-coated 96-wellplate with 1,5% of agar to form spheroids. 72 hours later after transduction, normal SCC13 or mock vs. CD271 spheroids were implanted in a matrix of human dermal fibroblasts-collagen type I solution composed as follows: DMEM, 200nM L-glutamine, 10% FBS, 7.5% sodium bicarbonate, 3mg/ml type I collagen previously extracted from rat tails and 1% penicillin/streptomycin supplemented with 15*10^4^/ml of primary human fibroblast derived from skin biopsies. Mock and CD271 spheroids were monitored each day until 1weeks, while normal SCC13 spheroids were treated with βAmyloid (40 or 60 μM) and monitored until 2 weeks or treated with the Trk inhibitor K252 (100 nM, 200 nM, 400 nM), Cisplatin (5 μg/ml) and 5-Fluoruacil (5FU) (50 μg/ml) for 48 hours.

**Expression profiling, computational analysis and accession number**

RNA sequencing (RNASeq) was performed on Mock vs. CD271 SCC13 spheroids in the NIAMS Genome Core Facility at the NIH (USA). NEBNext Ultra II protocol and kit were used to generate the RNASeq library and the HiSeq 2000 was used for sequencing. Significantly affected genes (p<0.05 and fold change 1.5) were determined by ANOVA. RNA-Seq expression fold changes were calculated using Partek Genomics Suite (http://www.partek.com). Pathway enrichment of significantly modulated genes (q<0.05 and Max RPKM40) was generated through Gene Ontology (GO) Term Enrichment using PANTHER gene list analysis tools (Mi et al., 2019). RNAseq raw data have been deposited in the Gene Expression Omnibus (GEO) site with the accession number GSE178845.

**Cell invasion assay**

Cell invasion assay was performed by seeding THP-1 cells in three different conditions, as schematized in Fig. S10a. Briefly, SCC13 cells were seeded in a 12-well plate (2*10^5^ cells/well) and infected with mock or CD271 viral vectors, as above described. After 48 hours, THP-1 cells (2*10^6^ cells/well), maintained in DMEM medium (DMEM (Dulbecco’s Modified Eagle Medium**),** supplemented with 10% FBS, 2% L-Glutamine, and 1% PSA), were added to the upper compartment of a 12-well Transwell chamber coated with Matrigel. Simultaneously, mock or CD271 SCC13-derived supernatants were added or not to the THP-1 cell suspension. In the lower compartment, transduced SCC13 cells underwent medium replacement with SCC medium containing 1% of FBS. After 48 hours, the supernatant, with or without the THP-1 cells, in the upper compartment was removed and Matrigel was dissolved with Collagenase Mix (500 U/ml Collagenase I and 200 U/ml Collagenase IV, Gibco), and infiltrating THP-1 cells were counted by using Blue-Trypan.

**Scratch assay**

# THP-1 cells were seeded in a 12-well plate (2*10^6^ cells/well) in DMEM medium. After 24 hours from seeding, cells were treated with the 12-O-Tetradecanoylphorbol-13-Acetate (TPA) (100 ng/ml; Sigma-Aldrich, Missouri, USA) to obtain cell priming and differentiation, as previously described (Baxter et al., 2020 J Immunol Methods). Subsequently, THP-1 cells were treated with Mitomycin C (10 μg/mL, Sigma-Aldrich, Missouri, USA) for 2 hours at 37°C and the wound was made by dragging a plastic pipette tip across the plate surface. The remaining cells were washed out with PBS. Finally, THP1 cells were treated with Mock or CD271 SCC13–derived supernatants containing 1% of FBS. Migrating cells at the wound front were photographed and counted at 48 hours from treatments.

**THP-1 cell immunofluorescence**

THP-1 cell differentiation into macrophages, after 24 hours stimulus with TPA and then with mock or CD271 supernatant, was confirmed by immunofluorescence staining for CD68. Before plating, THP1 cells were fixed with PFA 4% and subjected to cytospin in order to be used as a control. After treatments, cells were fixed with PFA 4% for 20’ at 4°C. Subsequently, cells underwent PBS- washes and the incubation with the primary antibody, reported in Supplementary Table 2, was performed overnight at 4°C. Incubation with the secondary antibody was performed for 45’ at room temperature. DAPI was used for nuclear staining. Micrographs were taken on a Confocal Scanning Laser Microscopy (Leica TCS4D, Leica, Exton, PA, USA). Positive cells vs total cells were counted for at least three different fields for each replicate.

**Supplementary Table 1**

|  | Forward primers | Reverse primers |
| --- | --- | --- |
| *hb-actin* | TGG ATG ATG ATA TCG CCG CGC TCG | CAC ATA GGA ATC CTT CTG ACC CA |
| *hNGFR* | TGA GTG CTG CAA AGC CTG CAA | TCT CAT CCT GGT AGT AGC CGT |
| *hNTRK1* | GGC TCC TCG GGA CTG CGA TG | CAG GAG AGA GAC TCC AGA GCG |
| *hNTRK3* | GGA AAG GTC TTC CTG GCC GAG TGC | GCT TTC CAT AGG TGA AGA TCT CCC |
| *hNGF* | TCA TCA TCC CAT CCC ATC TT | CTT GAC AAA GGT GTG AGT CG |
| *hBDNF* | AGC CTC CTC TTC TCT TTC TGC TGG A | CTT TTG TCT ATG CCC CTG CAG CCT T |
| *hNT-3* | TTT CTC GCT TAT CTC CGT GGC ATC C | GGC AGG GTG CTC TGG TAA TTT TCC T |
| *hNT-4* | ATG CTC CCT CTC CCC TCA T | GCA TGG GTC TCA GGC CCG |
| *hBIRC5* | GCATGGGTGCCCCGACGTTG | GCTCCGGCCAGAGGCCTCAA |
| *hCXCL8* | GAATGGGTTTGCTAGAATGTGATA | CAGACTAGGGTTGCCAGATTTAAC |

**Supplementary Table 2:** List of primary antibodies used in the study.

| Antibody | Provider | Dilution | Application |
| --- | --- | --- | --- |
| Mouse Anti-human CD271 | Millipore | 1:1000 | WB |
| Rabbit anti-human CD271 | Promega | 1:100 | IF |
| Rabbit anti-human TrkA | Invitrogen | 1:100/1:1000 | IF/WB |
| Rabbit anti-human pERK1/2 | Cell Signaling technology | 1:1000 | WB |
| Mouse anti-human p-p38 | Cell Signaling technology | 1:1000 | WB |
| Mouse anti-human β-actin | Sigma-Aldrich | 1:5000 | WB |
| Rabbit anti-human KRT15 | Abcam | 1:500 | IF |
| Rabbit anti-human KRT10 | Abcam | 1:500 | IF |
| Rabbit anti-human KRT1 | BioLegend | 1:500 | IF |
| Rabbit anti-human KRT13 | Abcam | 1:100/1:1000 | IF/WB |
| Rabbit anti-human Ki67 | Bethyl Laboratories, Inc | 1:100 | IF |
| Rabbit anti-Cyclin D1 | Roche | 1:100 | IHC |
| Rabbit anti-human Slug | Cell Signaling technology | 1:1000 | WB |
| Mouse anti-human Snail | Cell Signaling technology | 1:1000 | WB |
| Mouse anti-human E-Cadherin | BD Bioscience | 1:1000 | WB |
| Mouse anti-human CD68 | Roche | Ready to use | IF |

**Supplementary Table 3**

**Supplementary Figures**

**Fig. S1: CD271 expression in cSCC of different stages. a)** CD271 mRNA expression in cSCC of different stages from GSE45216 dataset analyzed by GEO2R **b)** Graphical representation of immunohistochemistry and immunofluorescence staining score of CD271, TrkA, Cyclin D1, KRT1 and KRT13 in cSCC biopsies of different stage of aggressiveness (AK, in situ, WD and MD/PD). **c)** Expression levels of KRT13, KRT10 and E-Cadherin protein in patient-derived spheroids of different stages determined by densitometry by using ImageJ software analysis. β-actin was used as loading control and normalizing protein. **d)** NTs and NTRs mRNA expression evaluated in patient-derived spheroids by qPCR. β-actin was used as a housekeeping gene. For all experiments, the results are shown as mean ± SD of three independent experiments. Statistical analysis was performed using the two-way ANOVA and multiparametric T-test. *0.01<*p* < 0.05, **0.001<*p* < 0.01, ***0.0001<*p* < 0.001, *****p* < 0.0001.

**Fig. S2**: **Western Blot densitometry, Real-time PCR and SCC12B and SCC15 spheroids evaluation. a)** Expression levels of E-Cadherin, KRT10 and Slug in mock vs CD271 overexpressing patient-derived spheroids measured by ImageJ software. β-actin was used as loading control and normalizing protein. **b)** NTRs and NTs mRNA relative quantification evaluated in SCC13, SCC12B and SCC15 spheroids by Real-Time PCR. β-actin was used as housekeeping gene. **c)** CD271-overexpressed or **d**) CD271-silenced SCC12B and SCC15 spheroids. Total area was measured by ImageJ software and cells viability was evaluated by MTT assay. For all experiments, the results are shown as mean ± SD of three independent experiments. Statistical analysis was performed using the two-way ANOVA and multiparametric T-test. *0.01<*p* < 0.05, **0.001<*p* < 0.01, ***0.0001<*p* < 0.001, *****p* < 0.0001.

**Fig. S3 Western blot quantification, and RNAseq analysis. a)** Expression levels of CD271, E-Cadherin, KRT10, ERK1/2, pERK1/2 and Slug in mock vs CD271 overexpressing or scramble vs CD271 siRNA SCC13 spheroids measured by ImageJ software. β-actin was used as loading control and normalizing protein. **b)** Volcano plot representing the most significant modulated genes between mock and CD271 SCC13 spheroids. For all experiments, the results are shown as mean ± SD of three independent experiments. Statistical analysis was performed using the two-way ANOVA and multiparametric T-test. **0.001<*p* < 0.01, *****p* < 0.0001.

**Fig. S4: Immunofluorescence quantification in CD271 vs Mock SCC13 spheroids and skin equivalent.** **a)** Expression level of CD271, TrkA, KRT1 and Ki67 in mock vs CD271 overexpressing SCC13-derived spheroids quantified by ImageJ. **b)** Epidermal thickness evaluation and expression level of CD271, KRT10 and KRT15 in mock vs CD271 SCC13 skin equivalent quantified by ImageJ. For all experiments, the results are shown as mean ± SD of three independent experiments. Statistical analysis was performed using the two-way ANOVA and multiparametric T-test. *0.01<*p* < 0.05, **0.001<*p* < 0.01, ***0.0001<*p* < 0.001.

**Fig. S5 CD271 expression abolishes cSCC metastasis in zebrafish model. a**) Schematic representation of the zebrafish experiment. **b)** Evaluation of zebrafish viability. **c)** Cake graphs showing the % of zebrafish with Full metastasis, Initial metastasis or In place cells after injection of mock vs CD271 overexpressing SCC13 and siRNA scramble and CD271-silenced SCC13 cells (n=25 zebrafish/condition) **d)** Upper panels: Densitometry of CD271, E-Cadherin, ERK1/2, pERK1/2, p-p38, Slug and Snail in cell injected into zebrafish. Β-actin was used as loading control. Bottom panel: pERK/ERK expression level ratio. Results are shown as the mean percentage of three independent experiments and a minimum of 20 injected zebrafish for each condition was used. Statistical analysis was performed using the two-way ANOVA and multiparametric T-test. *0.01<*p* < 0.05, ***0.0001<*p* < 0.001, *****p* < 0.0001.

**Fig. S6 CD271 activation decreases viability, invasion, and induces apoptosis in SCC spheroids. a)** Cell viability by MTT assay of mock vs CD271-overexpressing SCC12B spheroids after β-Amyloid (40 μM and 60 μM) treatment as compared to control (PBS). **b**) Total area of mock vs CD271-overexpressing SCC12B spheroids after β-Amyloid (40 μM and 60 μM) treatment as compared to control (PBS). **c)** Left panel: Propidium Iodide (PI) staining (red) on SCC12B spheroids after β-Amyloid (40 μM and 60 μM) treatment as compared to control (PBS). Right: Bar chart representing PI positive cells/total spheroid area ratio at 48 hours. **d**) Total area of SCC13-derived implanted spheroid treated with β-Amyloid (40 μM and 60 μM) or PBS (control). **e**) Total area of K252a (200 nM) vs DMSO (control) treated CD271-overexpressing or mock spheroids at 48 hours. For all experiments, the results are shown as mean ± SD of three independent experiments (at least n= 8 spheroids/condition/experiment). Statistical analysis was performed using the two-way ANOVA and multiparametric T-test. *0.01<*p* < 0.05, **0.001<*p* < 0.01, ***0.0001<*p* < 0.001, *****p* < 0.0001.

**Fig. S7 CD271 activation increases the response to chemotherapy and promotes macrophage recruitment in zebrafish.** **a**) Schematic representation of the zebrafish experiment**. b)** Evaluation of zebrafish viability. **c)** Cake graphs showing the % of zebrafish with Full metastasis, Initial metastasis or In place cells after treatment with Cisplatin (5 μg/mL), β-Amyloid (40 μM) or a combination, as described. PBS was used as control. Results are shown as the mean percentage of three independent experiments and a minimum of 20 injected zebrafish for each condition was used.

**Fig. S8 CD271 activation promotes macrophage recruitment in zebrafish. a)** and **b)** Representative images of *mpeg1*-positive cells (red) in zebrafish injected with mock or CD271-overexpressing cells (green) at 5 dpi (n= 3, 3 independent evaluations). a) Scale bar= 500 μm and b) Scale bar= 100 μm

**Fig. S9 CD271 activation promotes macrophage recruitment in zebrafish. a)** Representative images of *mpeg1* whole-mount in situ hybridization in zebrafish injected with mock or CD271-transduced SCC13 cells or **b)** after treatment with Cisplatin (5 μg/ml) or PBS (control) and β-Amyloid (40 μM). Positive *mpeg1* cells were indicated with white arrows. **c)** and **d)** Representative mask images of *mpeg1* whole mount in situ hybridization zebrafish analyzed with ImageJ software. Results are shown as the mean percentage of three independent experiments and a minimum of 20 injected zebrafish for each condition was used.

**Fig. S10 THP-1 invasion is increased by CD271 overexpressing SCC cells-derived supernatant a)** Schematic representation of the cell invasion assay performed by seeding THP-1 cells on Transwell support, in the presence of Matrigel, under three different conditions: 1) with mock or CD271 supernatant, without cSCC cells in the lower compartment, 2) without mock or CD271 supernatant, with cSCC cells in the lower compartment, 3) with mock or CD271 supernatant, with cSCC cells in the lower compartment **b)** Quantification of the number of invading THP-1 cells. Results are shown as the mean percentage of three independent experiments. Statistical analysis was performed using the two-way ANOVA and multiparametric T-test. **0.001<*p* < 0.01, *****p* < 0.0001.

**Fig. S11 THP-1 migration is increased by CD271 overexpressing SCC cells-derived supernatant** a) Evaluation of CD68 expression in THP1 cells after TPA and mock or CD271 supernatant stimulus b) Representative images ad quantification of scratching assay on THP-1 cells treated with TPA (100 ng/ml) and stimulated with mock or CD271 overexpressing cSCC cells-derived supernatants. The invasion area of THP-1 was measured by ImageJ software. Results are shown as the mean percentage of three independent experiments. Statistical analysis was performed using the two-way ANOVA. *0.01<*p* < 0.05, **0.001<*p* < 0.01, ***0.0001<*p* < 0.001.
